# Preventive effects of the *Rehmannia glutinosa* Libosch and *Cornus officinalis* Sieb herb couple on chronic kidney disease rats *via* modulating the intestinal microbiota and enhancing the intestinal barrier

**DOI:** 10.3389/fphar.2022.942032

**Published:** 2022-09-08

**Authors:** Ling Wang, Jin-Hui Zhu, Xiao-Dan Jiang, Zhen-Xiang Ma, Jin-Hua Tao

**Affiliations:** ^1^ School of Public Health, Nantong University, Nantong, China; ^2^ School of Pharmacy, Nantong University, Nantong, China; ^3^ Department of Occupational Medicine and Environmental Toxicology, Nantong Key Laboratory of Environmental Toxicology, Nantong University, Nantong, China

**Keywords:** chronic kidney disease, adenine, *Rehmannia glutinosa* Libosch, *Cornus officinalis* Sieb, intestinal microbiota, barrier function

## Abstract

CKD is a clinical syndrome with slow development and gradual deterioration of renal function. At present, modern medicine still lacks an ideal treatment method for this disease, while TCM has accumulated rich clinical experience in the treatment of CKD, which can effectively improve renal function and delay renal failure, and has unique advantages. RC is widely used in clinical practice to treat CKD, especially the “Kidney-Yin” deficiency syndrome. However, the compatibility mechanisms responsible for its effects in experimental studies, including preclinical and clinical research studies, are still not fully understood. Adenine-induced CKD rats were used to investigate the preventive effect of RC on CKD rats. Based on the high-throughput 16S rRNA gene sequencing results from Illumina, we discussed the intestinal flora abundance in rats in different treatment groups. According to a PCA and a PCoA based on a distance matrix, there was a clear separation of gut microbiome profiles between normal rats and model rats in terms of beta diversity. The abundance of Firmicutes in CKD rats was relatively increased, while that of Bacteroidetes was decreased. It is clear that the plot for the RC group was closer to that of the normal group, suggesting that the RC group had higher similarities among bacterial members with N rats. Ussing chamber, Western blot, and PCR assays were used to investigate the effects of RC on intestinal barrier function and its molecular mechanism in model animals. The results indicated that the protein expressions of ZO-1, claudin-1, and occludin-1 were decreased significantly in chronic kidney disease rats with the induction of adenine. With the treatment of RG, CO, and RC, the intestinal barrier was repaired due to the upregulated expressions of the aforementioned proteins in CKD rats. Based on our findings, RC appears to strengthen the intestinal barrier and modulate gut microbiota in adenine-induced CKD rats. This project revealed the compatibility mechanism of RC in regulating the intestinal microecology and barrier function to intervene in CKD and provided the basis and ideas for the clinical application of RC and the development of innovative drugs for CKD.

## 1 Introduction

Chronic kidney disease (CKD) is a clinical syndrome with slow development and gradual deterioration of renal function. The incidence of CKD in our country exceeds 10%, and it has become a worldwide health problem carrying relevant medical, social, and economic consequences (Ioannidou et al., 2011; Zhang et al., 2012; Zha et al., 2020). For human health and life, delaying or blocking the progression of CKD has become an important challenge faced by the clinical medical community and the health departments of various countries. At present, modern medicine still lacks an ideal treatment method for this disease, whereas traditional Chinese medicine (TCM) has amassed extensive clinical experience treating it, which can effectively improve renal function and delay renal failure, and has unique advantages. However, due to the complex formulation of traditional Chinese medicine, it is difficult to determine its pharmacodynamic mechanism. Fortunately, as a specific combination of two relatively fixed TCMs, drug pairs are not only the simplest and most basic forms of TCM formulations but are also synergistic (Qu et al., 2020). The study of herb–herb interactions has gained more attention over the last few decades as research has confirmed that herb pairs have a better pharmacological efficacy than individual herbs (Wang et al., 2016). The study of drug pairs makes it easier to elucidate the compatibility issues of complex TCM formulations. Therefore, the study of herbal pair interactions has received great attention.

*Rehmannia glutinosa* Libosch (RG) mainly contains the major components of iridoids (e.g., catalpol) and phenylethanol glycosides (e.g., verbascoside), is clinically beneficial to the liver and kidney, and is applied to promote circulation of the blood. *Cornus officinalis* Sieb (CO) mainly contains iridoid glycosides (e.g., mononoside and troside) and has been used to rejuvenate the liver and kidneys, reduce urination, and maintain kidney essence in traditional Chinese medicine (TCM) formulations for thousands of years. The combination of *Rehmannia glutinosa* and *Cornus officinalis* comes from the famous Chinese medicine prescription “Liuwei Dihuang Pill.” Because of its significant effect of “nourishing yin and invigorating the kidney,” it has been widely used by doctors of all dynasties. Many classic prescriptions such as Jisheng Shenqi pill and Zuogui pill contain this drug pair, and it is also a combination of herbs which is used to treat “Kidney-Yin” deficiency syndrome (Wang et al., 2013; Tao et al., 2018). In this study, our research group used the established “Traditional Chinese Medicine Prescription Database Retrieval System” (derived from the “Chinese Medicine Prescription Dictionary”) to search for prescriptions related to chronic kidney disease in the main diseases and used the bibliographic information co-occurrence analysis system (Bibliographic Item Co-Occurrence Matrix Builder) as a data processing tool to statistically analyze the frequency of occurrence of each traditional Chinese medicine in the prescriptions for chronic kidney disease. The results showed that the application frequency of *Rehmannia glutinosa* and *Cornus officinalis* was significantly higher, and the frequency of co-occurrence of the two medicines was also higher. In addition, *Rehmannia glutinosa* and *Cornus officinalis* had the highest ratio of 2:1 in the prescriptions for treating chronic kidney disease, accounting for 70% of the total. Although RC is a well-known prescription for CKD in traditional medicines, the compatibility mechanisms responsible for its effects in experimental studies, including preclinical and clinical research studies, are still not fully understood.

In humans, the gut is a symbiotic system with a large number of bacteria and microorganisms, which constitute an extremely complex micro-ecosystem and maintain a relatively stable balance, affecting the immune anti-inflammatory axis and playing an important role in maintaining human health (Holmes et al., 2012; Nicholson et al., 2012; Valentina and Fredrik, 2012). Cumulative evidence has demonstrated a crucial role of intestinal microbiota in the pathogenesis of chronic kidney diseases (Pan and Kang, 2017; Feng et al., 2019; Plata et al., 2019). CKD patients usually suffer from dysbiosis of gut microbiota, resulting in a changed composition of the intestinal flora. As a result, this altered colonic flora produces uremic toxins, such as indoxyl sulfate and p-cresyl sulfate, that accumulate in the body (Zhang et al., 2012; Crespo-Salgado et al., 2016; Wanchai et al., 2017), which may contribute to progression of chronic renal disease, and are normally excreted in the urine (Gholamreza et al., 2020). At the same time, intestinal microecological disturbances lead to the proliferation of intestinal pathogens, resulting in the production of a large amount of endotoxin, which damages the intestinal mucosal barrier, increases intestinal permeability, and leads to the translocation of intestinal bacteria and endotoxins to other tissues. This causes intestinal infection and endotoxemia, and endotoxin activates transcription factors NF-κB and AP1 through Toll-like receptor 4 (TLR4) and promotes inflammatory cytokines such as TNF-α, IL-1, IL-6, and MCP-1. These factors ultimately cause systemic inflammatory response and accelerates the process of CKD and aggravates kidney damage (Ramezani and Raj, 2014; Shiba et al., 2014; Vanholder and Glorieux, 2015). Therefore, strategies for the kidney–gut axis aimed at altering gut microbiota composition and rebuilding the gut barrier, including supply of prebiotics and/or probiotics, have been proposed for CKD.

In this study, potentially protective effects of the *Rehmannia glutinosa* Libosch and *Cornus officinalis* Sieb herb couple on adenine-induced CKD rats were evaluated systematically through macroscopical scores, histological changes, and inflammatory markers. Furthermore, the modulation of intestinal microflora by the herb couple was examined by Illumina MiSeq sequencing of 16S rRNA gene libraries *via* high-throughput sequencing. In addition, the intestinal barrier function-related gene expression analysis was also carried out to explore the potential biological mechanism of *Rehmannia glutinosa* Libosch and *Cornus officinalis* Sieb herb couple’s intervention in chronic kidney disease. This study has important implications for exploring new strategies for the clinical treatment of chronic kidney disease based on gut microecology.

## 2 Materials and methods

### 2.1 Animals

A total of sixty male Sprague–Dawley rats weighing between 180 and 220 g were obtained from Vital River Laboratory Animal Technology Co., Ltd. (Beijing, China). The experimental animal license number was SCXK (Su)-2016-0003. Approval for these experimental protocols was obtained from the Animal Research Committee of Nantong University (Animal Ethics Number. 20200826-003), and all animals were treated according to guidelines laid out by the National Institutes of Health. The rats were maintained on a 12:12 light/dark cycle at a room temperature of 22 ± 2°C and a relative humidity of 50 ± 10%.

### 2.2 Chemical regents

Adenine (Nanjing Herbal Biotechnology Co., Ltd., batch number BZP 1910), Huangkui capsules (Jiangsu Suzhong Pharmaceutical Group Co., Ltd., batch number 59288285688), *Cornus officinalis* (Sanyue Traditional Medicine, batch number 200704), and *Rehmannia glutinosa* crude material (Sanyue Traditional Medicine, batch number 200627) were procured.

The BUN, SCr, and UCR ELISA kits were purchased from Nanjing Herbal Biotechnology Co., Ltd., Nanjing, China. In addition, Rat UP ELISA Kit (Nanjing Herbal Biotechnology Co., Ltd., batch number 202009), Rat TGF-β ELISA Kit (Nanjing Herbal Biotechnology Co., Ltd., batch number 2105R21), Rat IFN-γ ELISA Kit (Nanjing Herbal Biotechnology Co., Ltd., batch number 2105R33), and Rat IL-6 ELISA Kit and Rat TNF-α ELISA Kit (Meimian Biotechnology Co., Ltd., batch number MM-0190R2, and MM-0180R2) were also procured for the study.

The following materials were obtained for the study: polyvinylidene fluoride (PVDF) (Beyotime Biotechnology, batch number FFP28), Prestained Color Protein Molecular Weight Marker (Beyotime Biotechnology, batch number P0071), RIPA lysis buffer (Beyotime Biotechnology, batch number P0013B), phenylmethanesulfonyl fluoride (PMSF) (Beyotime Biotechnology, batch number ST506), Enhanced BCA Protein Assay Kit (Beyotime Biotechnology, batch number P0010), Dual Color SDS-PAGE Protein Sample Loading Buffer, 5X, odorless (Beyotime Biotechnology, batch number P0295-15ml), blotting grade (Beyotime Biotechnology, batch number P0216-1500g), β-Actin (13E5) Rabbit mAb (1:2000, Cell Signaling Technology, batch number 4970S), monoclonal rabbit anti-occludin antibody (1:1000, Abcam, batch number ab167161), anti-claudin-1 antibody (1:1500, Abcam, batch number ab15098), ZO-1 rabbit polyclonal antibody (1:1000, Proteintech, batch number 21773-1-AP), Alexa Fluor^®^ 680-AffiniPure Goat Anti-Rabbit IgG (H+L) (Jackson, batch number 111-625-144), HiScript III RT SuperMix for qPCR (+gDNA wiper) (Vazyme, batch number R333-00 100 rxns), and ChamQ SYBR^®^ qPCR Master Mix (High ROX Premixed) (Vazyme, batch number Q341-02).

### 2.3 Experimental procedures

#### 2.3.1 Adenine-induced kidney disease in rats

After an acclimatization period of 7 days, the rats were fed with standard chow and water *ad libitum*; all rats were randomly distributed into six equal groups. The first group was the normal group (N group), the second group was the model group (M group), and the third, fourth, fifth, and the sixth were the therapy groups. Except for the rats in the normal group, all other rats were given adenine suspension (freshly dissolved in 0.5% CMC–Na) intragastrically for 14 consecutive days to establish a model of CKD induced by adenine. The rats in the normal group were given 0.5% sodium carboxymethyl cellulose aqueous solution every day. The third group was treated orally with Huangkui capsules (the HK group). Here, an aqueous solution of Huangkui capsule powder dissolved in ultrapure water was prescribed, with a daily dose of 675 mg/kg, which is equivalent to 7,500 mg of Huangkui capsules per day for patients weighing 60 kg. The rats in the fourth, fifth, and the sixth groups were treated with the *Rehmannia glutinosa* extract (the RG group), *Cornus officinalis* extract (the CO group), and *Rehmannia glutinosa* Libosch and *Cornus officinalis* Sieb herb couple extract (RC group). Experimental protocols for animal care and welfare were strictly in accordance with the Guide for the Care and Use of Laboratory Animals.

#### 2.3.2 Preparation of the biological sample

Fresh stool samples were collected from each rat after the treatment on the 14th day, frozen immediately, and stored until processing at −80°C. All rats were killed using deep ether anesthesia, and then, their colons were dissected longitudinally along a longitudinal mesentery. They were further rinsed with isotonic saline and assessed for intestinal mucosa. The kidney tissues were also collected for analyses.

In a 24-h period after the end of the treatment, the rats were anesthetized by intraperitoneal injection of chloral hydrate. Approximately, 6 ml of blood was collected from the abdominal aorta and centrifuged at 1,200 g at 4°C for 10 min to separate the plasma. In preparation for analysis, the plasma was stored at −80°C. Both kidneys were removed, blotted dry on a filter paper, and weighed. The relative kidney weight was determined by multiplying the kidney weight times the body weight. We rapidly preserved a little portion of the left kidney in formalin saline in preparation for histopathological and immunohistochemical processing. The remaining kidney tissues were frozen at −80°C for subsequent biochemical testing.

#### 2.3.3 Ussing chamber experiment

The prepared intestinal segments and intestinal mucosa of the test animals in each group were fixed in the diffusion chamber; the mucosal side was washed with Ringer’s solution (pH = 7.3) (Li et al., 2021; Stevens et al., 2021), and the biological activity of the intestinal mucosa was maintained at a constant temperature of 37.5°C for 30 min. Transepithelial resistance was calculated after the equilibration period, and TER (Ω cm^2^) was recorded every 15 min for 1 h. Data were averaged to calculate the TER value for each rat. The current signals of changes in the ion channels of the entire cell membrane were detected using microelectrodes, reflecting changes in intestinal drug absorption, permeability, and secretion (Chen et al., 2015).

#### 2.3.4 Enzyme-linked immunosorbent assay

The Tris-HCl containing protease inhibitors were used to extract colon tissue with appropriate amounts of guanidine hydrochloride. Then, the extract was centrifuged (13,000 g × 10 min) at 4°C, and subsequently, the ELISA kit was used to determine the cytokines in the supernatant, as directed by the manufacturer.

#### 2.3.5 Intestinal barrier function-related gene expression analysis

The TRIzol method was used to extract total RNA from the intestinal mucosa samples, according to the instructions provided by the total RNA extraction kits. The total RNA level was studied using a spectrophotometer with a wavelength of 260 nm. The integrity and quality of the extracted RNA was analyzed using 1% agarose gel electrophoresis and the absorbance ratio at 260/280 nm. For reverse transcription, total RNA was extracted from intestinal mucosa samples, as directed by the RT reagent kit (TaKaRa Biotechnology Co., Ltd.).

The real-time quantitative reverse-transcription (qRT)-PCR was used to determine the three tight junction (TJ) protein genes: occludin-1, claudin-1, and the encoding zonula occludens 1 (ZO-1) gene expression level of the intestinal mucosa. According to the NCBI database (http://www.ncbi.nlm.nih.gov/), the gene sequences of claudin-1, occludin-1, and ZO-1 were obtained, using this gene as a template, and then, according to the principle of primer design, with the assistance of Oligo Design software, primers for claudin-1, occludin-1, ZO-1, IL-4, and IL-6 genes were obtained (Table 1). All primers were confirmed by NCBI BLAST software for their specificity. Each primer sequence was synthesized by Invitech (Shanghai) Trading Co., Ltd. The relative mRNA expression of the target genes was determined using the 2^−ΔΔCT^ method, and gene relative expression levels were expressed as a fold change to the internal control gene β-actin.

**TABLE 1:** Designed primer sequence.

Primer name	Primer sequence (5′--3′)
Occludin-1	TCT​CTC​AGC​CGG​CAT​ACT​CT
	GCG​ATG​CAC​ATC​ACG​ATG​AC
ZO-1	ACA​GCC​AGC​TCT​TGG​TCA​TC
	GTA​TGG​TGG​CTG​CTC​AAG​GT
Claudin-1	TGG​CAT​GAA​GTG​CAT​GAG​GT
	CCC​AGC​CAG​TAA​AGA​GAG​CC
β-actin	AGG​GAA​ATC​GTG​CGT​GAC​AT
	GAA​CCG​CTC​ATT​GCC​GAT​AG
IL-6	CAC​TTC​ACA​AGT​CGG​AGG​CT
	TCT​GAC​AGT​GCA​TCA​TCG​CT
IL-4	TCC​AGG​GTG​CTT​CGC​AAA​T
	GTT​CAG​ACC​GCT​GAC​ACC​TC

#### 2.3.6 Western blot experiment

Colon tissues were ground and lysed with RIPA lysis buffer and phenylmethanesulfonyl fluoride (PMSF). The protein concentration was determined by using the BCA Protein Assay Kit. SDS-PAGE was used to separate the proteins, and the PVDF membrane was used to transfer those proteins. Then, they were blocked with phosphate-buffered saline with Tween 20 (PBST) containing 5% fat-free milk. After washing three times in TBST, PVDF membranes were co-incubated with the primary Abs (ZO-1, occludin-1, and claudin-1) overnight at 4°C and then incubated with secondary Abs (Goat Anti-Rabbit IgG) for 1 h at room temperature. After washing three times in TBST, photographs were taken with β-actin as the internal reference in the imaging system (LI-COR Odyssey).

#### 2.3.7 Intestinal bacterial gene amplification and Illumina MiSeq sequencing

Using the Omega Bio-tek EZNA^®^ Soil DNA Kit (Norcross, GA, United States), the microbial DNA was extracted from colonic contents. Amplificating the V3-V4 regions of the 16S rDNA from bacteria was achieved by PCR (95°C for 4 min, followed by 25 cycles of 30 s each at 95, 55, and 72°C for 45 s, and then by one last extension of 10 min at 72°C) using primers 341F and 806R, which contain barcodes CCTAYGGGRBGCASCAG-3′ and GGACTACNNGGGTA TCTAA T-3′, respectively. Amplicons were extracted from 2% agarose gels using the AxyPrep DNA Gel Extraction Kit and quantified using the QuantiFluorTM-ST system. The pooled DNA products were used in Illumina’s genome DNA library preparation procedure to make Illumina pair-end libraries. In the following steps, the amplicon library was sequenced on an Illumina MiSeq platform (Shanghai BIOZERON Co., Ltd.), according to the standard protocols.

#### 2.3.8 Process of sequencing data

Dehybridization filtering was performed on the data prior to in-depth bioinformatic analysis of the sequencing results. By FLASH (v 1.2.7) software, according to the minimum overlap length of 10 bp, the maximum mismatch ratio allowed in the overlap region was 0.2, splicing the reads of each sample. The obtained splicing sequence is the original tag data. Trimmomatic (v0.33) software was used to filter the spliced raw tags to obtain high-quality tag data, and then, UCHIME (v 4.2) software was used to identify and remove chimera sequences to obtain the final effective data for subsequent bioinformatics analysis. QIIME (version 1.17) was used (Sun, Li et al., 2015) to demultiplex and filter the original FASTQ files. UPARSE version 7.1 was used to cluster OTUs. Chimeric sequences were verified and deleted by UCHIME. Based on the Silvia database, we evaluated the genetic relationships of each 16S rDNA sequence using the ribosomal database project classifier (http://rdp.cme.msu.edu/) (Westcott and Schloss 2015).

#### 2.3.9 Statistical analysis

A mathematical analysis of the experimental data was carried out by GraphPad PrismV5 software. An analysis of variance (ANOVA) with Tukey’s honestly significant difference (HSD) *post hoc* test was used to compare groups’ average number of members (Tao et al., 2018).

## 3 Results

### 3.1 Amelioration of RG, CO, and RC on adenine-induced chronic kidney disease in rats

**FIGURE 1:**
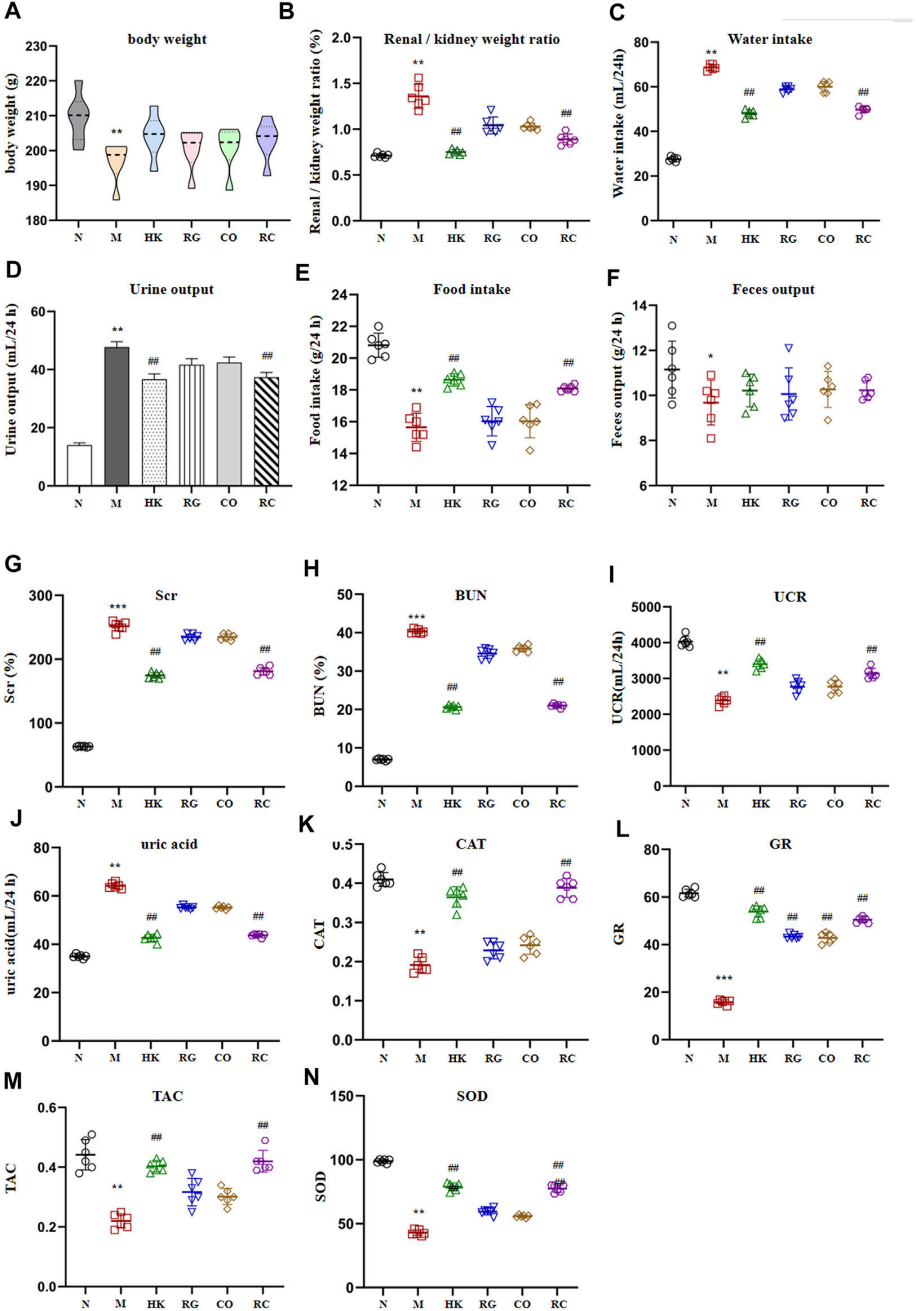
Intervention effect of RG, CO, and RC on some physiological parameters: **(A)** body weight (g), **(B)** renal/kidney weight ratio (%), **(C)** water intake (ml/24 h), **(D)** urine output (ml/24 h), **(E)** food intake (g/24 h), **(F)** fecal output (g/24 h), **(G)** SCr (%), **(H)** BUN(%), **(I)** UCR (ml/24 h), **(J)** uric acid (ml/24 h), **(K)** CAT, **(L)** GR, **(M)** TAC, and **(N)** SOD. (Data are means ± SD. ***p < 0.001, **p < 0.01, and *p < 0.05 vs*.* normal group; ^##^ *p <* 0.01, and ^#^ *p <* 0.05 vs*.* model group. *n* = 6).

Compared with the control group (N), the weight of adenine-induced chronic kidney disease rats (M) decreased continuously in 2 weeks of adenine modeling (Figure 1). Adenine-induced CKD rats not only lost weight but also significantly increased in the renal/kidney weight ratio, water intake, and urine output throughout the modeling period; meanwhile, the kidney index was significantly increased (*p* < 0.01), indicating oral administration of adenine led to renomegaly (Figure 1). Meanwhile, the food intake and fecal output were decreased obviously (*p* < 0.05). Treatment with *Rehmannia glutinosa* extracts (RG), *Cornus officinalis* extracts (CO), and the herb couple extracts (RC) did not induce notable change in body weight and fecal output, but RC treatment attenuated the decrease in the kidney weight ratio (*p* < 0.01).

A significant increase in SCr, BUN, and uric acid levels (*p* < 0.01) was observed in the model group after 2 weeks of adenine administration (Figure 1). RG, CO, and RC attenuated adenine-induced increases in all of the aforementioned parameters, with RC having the most significant intervention effect (*p* < 0.05). Figure 1 also shows that adenine significantly reduced CAT, TAC, GR, UCR, and SOD levels; RG or CO alone improved the aforementioned physiological parameters but have no significance. However, the reduction of CAT, TAC, GR, UCR, and SOD levels in the adenine-induced CKD rats by RC treatment was significantly ameliorated.

### 3.2 Pathological change in the adenine-induced chronic kidney disease rats

**FIGURE 2:**
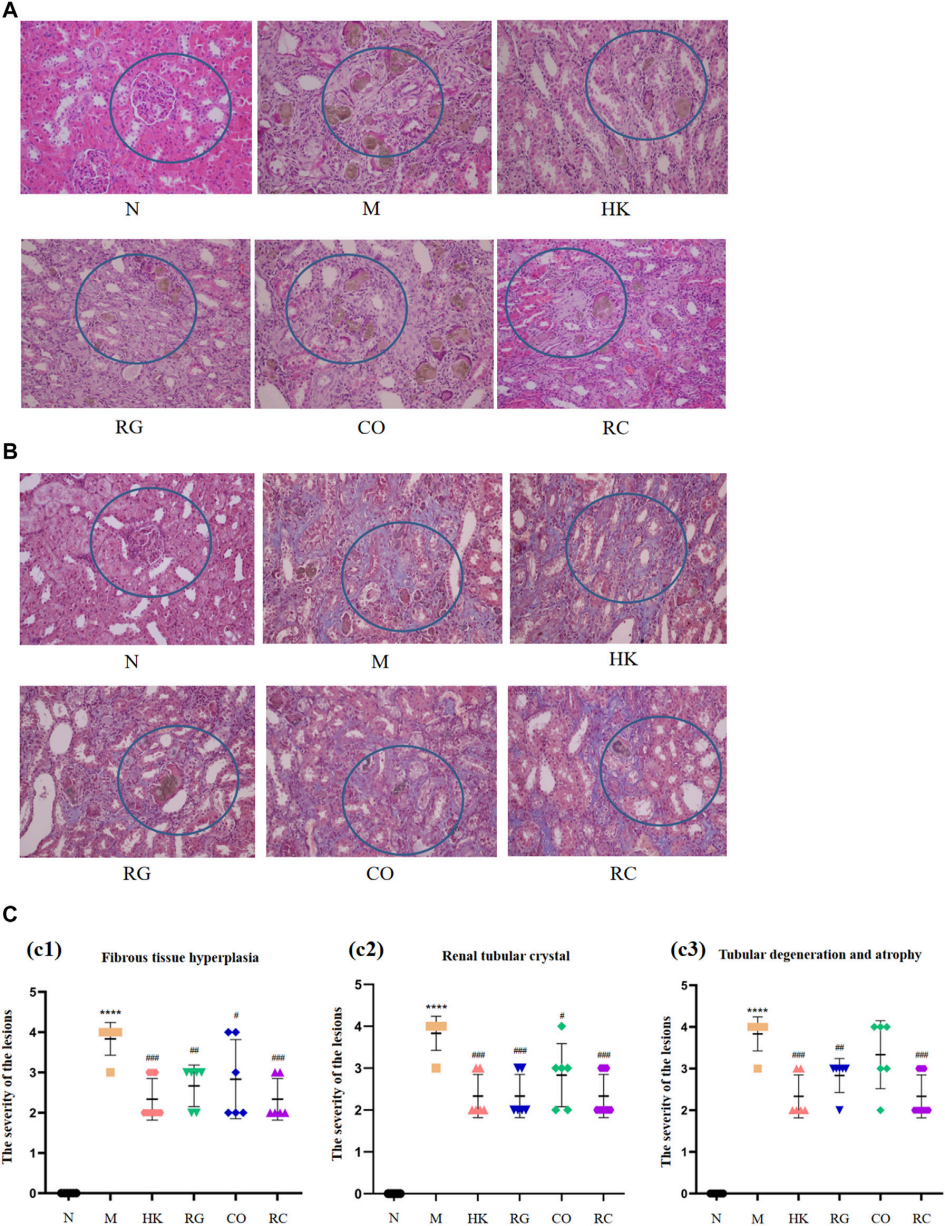
Routine histopathological examination and collagen deposition of the kidneys of rats from different groups were evaluated by different staining methods. **(A)** H&E staining of the kidney of rats. **(B)** Masson staining of the kidney of rats. **(C)** Histological scoring was obtained from the H&E and Masson staining results of the kidney (c1) Fibrous tissue hyperplasia, (c2) Renal tubular crystal, (c3) Tubular degeneration and atrophy. (Data are means ± SD. ****p < 0.0001 and **p < 0.01 vs*.* normal group; ^###^ *p <* 0.001, ^##^ *p <* 0.01, and ^#^ *p <* 0.05 vs*.* model group. *n* = 6).

The routine histopathological examination and demonstration of collagen deposition of the kidneys from different groups were evaluated by H&E staining (Figure 2A) and M&T staining (Figure 2B), respectively. By observing microscopic images of histopathology and the semi-quantitative score (Figure 2C), it can be seen that the control rats (N) displayed normal architecture and histology of renal tissues with the aforementioned two stains. Compared with the control group, CKD rats exhibited tubular and glomerular atrophy, interstitial fibrosis, and interstitial inflammation in their renal tissues. The results further demonstrated that CKD rats induced by adenine were successful. Nevertheless, both the positive (HK) and the RC groups had remarkable improvements in symptoms. Additionally, collagen expression was evident in the interstitial and tubular regions of the kidneys of CKD rats. Compared with the model group, HK, RG, CO, and RC reversed the renal pathological damage caused by adenine to different degrees. The results of these experiments indicate that RC is effective in reducing kidney injury in rats induced by adenine.

### 3.3 Gut barrier integrity function

**FIGURE 3:**
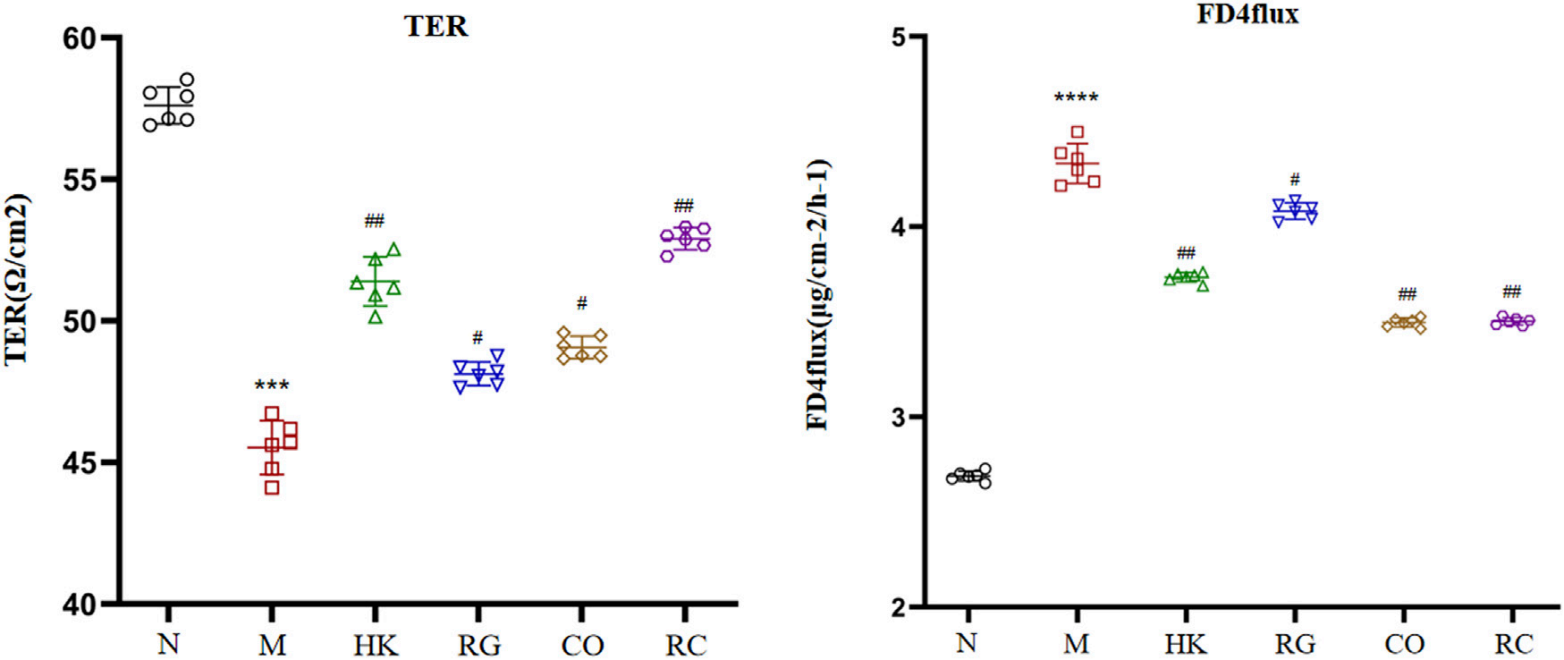
Indexes of the intestinal barrier function. (Data are means ± SD. ****p < 0.0001 and ***p < 0.001 vs*.* normal group; ^##^ *p <* 0.01 and ^#^ *p <* 0.05 vs*.* model group. *n* = 6).

The gut barrier function index (TER and FD4 flux across cells) is shown in Figure 3. In comparison with the normal rats, the transepithelial electrical resistance in colon tissue of the adenine-induced CKD rats was significantly reduced, and meanwhile, the fluorescein isothiocyanate–dextran 4 kDa flux was increased (*p* < 0.05). However, the *Rehmannia glutinosa* Libosch and *Cornus officinalis* Sieb herb couple extract increased the transepithelial electrical resistance in colon tissue and decreased fluorescein isothiocyanate–dextran 4 kDa fluxes in colon tissue of the adenine-induced CKD rats (*p* < 0.05). At the same time, the RG and CO groups had no effect (*p* > 0.05) in the aforementioned parameters.

### 3.4 Analysis of circulating cytokines in the serum

**FIGURE 4:**
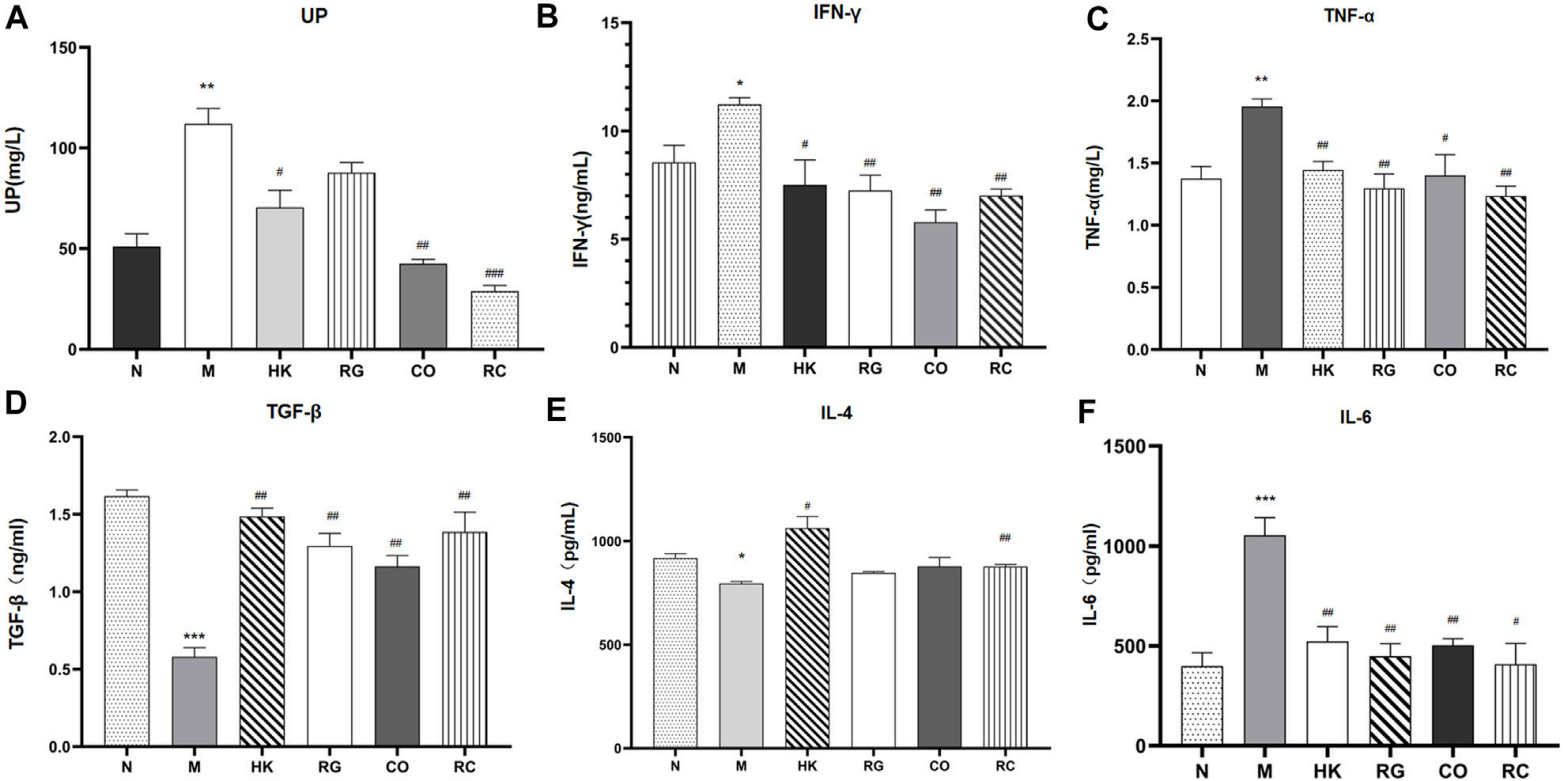
Concentration and expression of related biochemical cytokines. (Data are means ± SEM. **(A)** UP; **(B)** IFN-γ; **(C)** TNF-α; **(D)** TGF-β; **(E)** IL-4 ; **(F)** IL-6. ***p < 0.001 vs. normal group; **p < 0.01 and *p < 0.05 vs*.* normal group; ^##^ *p <* 0.01 and ^#^ *p <* 0.05 vs*.* model group. *n* = 6).

The concentration and expression of urinary protein (UP), IFN-γ, TNF-α, TGF-β, IL-4, and IL-6 were analyzed in the serum, respectively (Figure 4). Results consistently showed that the levels of the UP and classical pro-inflammatory cytokines IFN-γ, TNF-α, and IL-6 in adenine-induced CKD rats were significantly higher than those in other groups, while the levels of TGF-β and IL-4 in adenine-induced CKD rats were notably lower than those in the normal group. RG, CO, and RC significantly attenuated adenine-induced increase in the measured inflammatory markers. At the same time, the results also showed that the effect of the RC group was significantly better than that of RG or CO alone. The levels of IL-4 showed no statistical difference in the three groups after treatment (*p* > 0.05).

### 3.5 Barrier function-related gene expression

To further investigate the effects of RC on the intestinal barrier integrity, the protein levels and relative mRNA expressions of tight junction protein (TJP) including zonula occludens-1 (ZO-1), claudin-1, and occludin-1 were measured.

**FIGURE 5:**
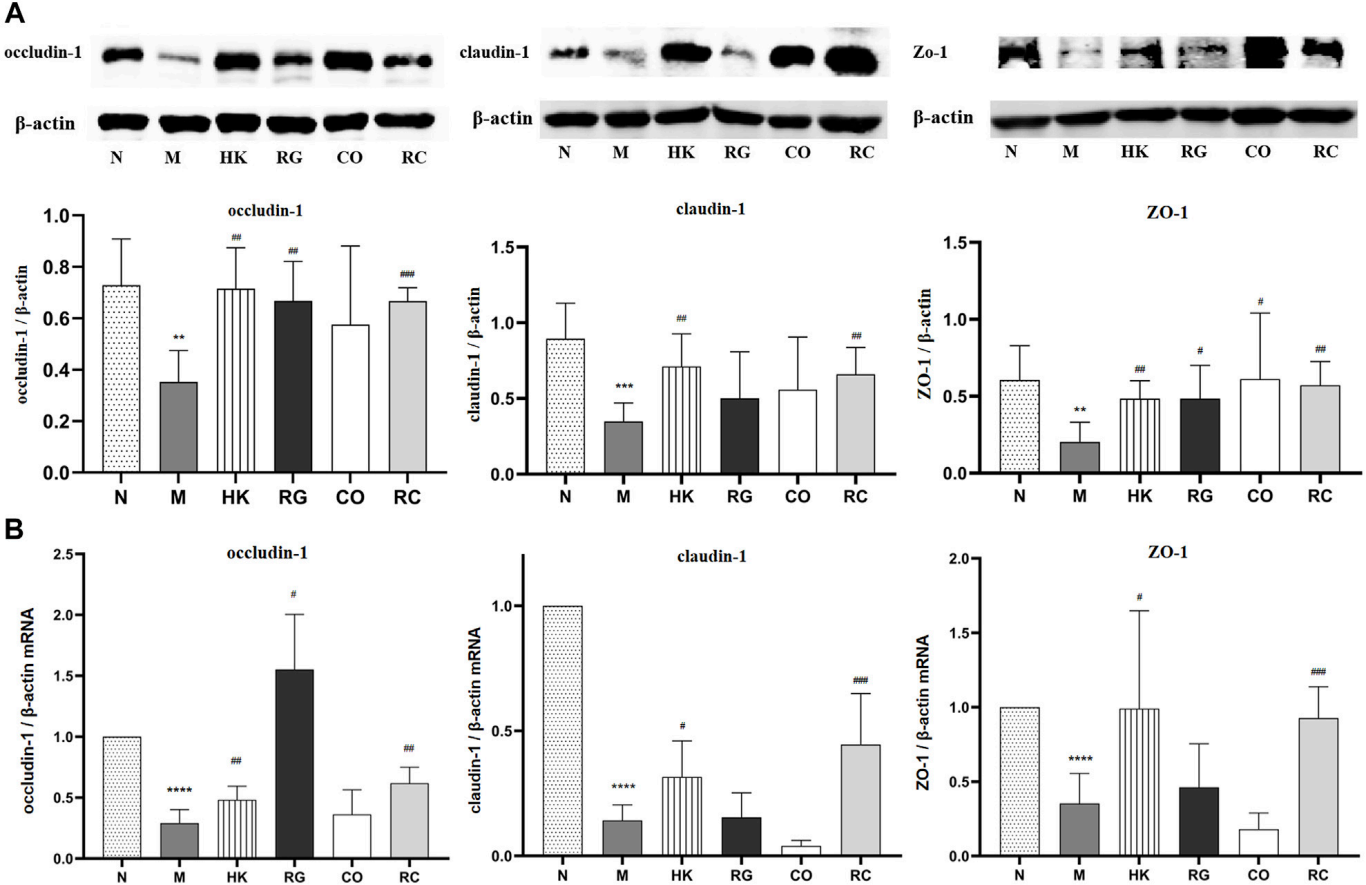
**(A)** Effects of RG, CO, and RC on the expressions of occludin-1, ZO-1, and claudin-1 in CKD rats. **(B)** Effects of RG, CO, and RC on the mRNA levels of occludin-1, ZO-1, and claudin-1 in CKD rats. (Data are means ± SD. ****p < 0.0001 vs*.* normal group; ^###^ *p <* 0.001, ^##^ *p <* 0.01, and ^#^ *p <* 0.05 vs*.* model group. *n* = 6).

We found that the protein expressions of the abovementioned proteins were decreased significantly in chronic kidney disease rats with the induction of adenine. With the treatment of RG, CO, and RC, the intestinal barrier was repaired due to the upregulated expressions of ZO-1, claudin-1, and occludin-1 of CKD rats (Figure 5A). Meanwhile, the mRNA expressions of such tight junction-related genes, which were tested by qRT-PCR, were also significantly decreased in CKD rats. Similarly, after RC treatment, the corresponding mRNA expression was significantly increased (Figure 5B).

### 3.6 Alpha and beta diversity of microbial 16S rRNA genes

To determine whether RG, CO, and RC were ameliorated in rats with adenine-induced CKD, 36 gut content samples were applied in this study. A high-throughput sequencing procedure was carried out on the Illumina MiSeq platform. In fewer than 97% identity conditions, there were 1,728,149 reads that passed all quality filters, resulting in 254,304 species classification OTUs. Generally, Shannon and Simpson indices are often used to describe the diversity of microbial communities in a particular community or ecosystem. The greater the Shannon index, the higher is the biodiversity; there is a negative correlation between the Simpson index and biodiversity.

**FIGURE 6:**
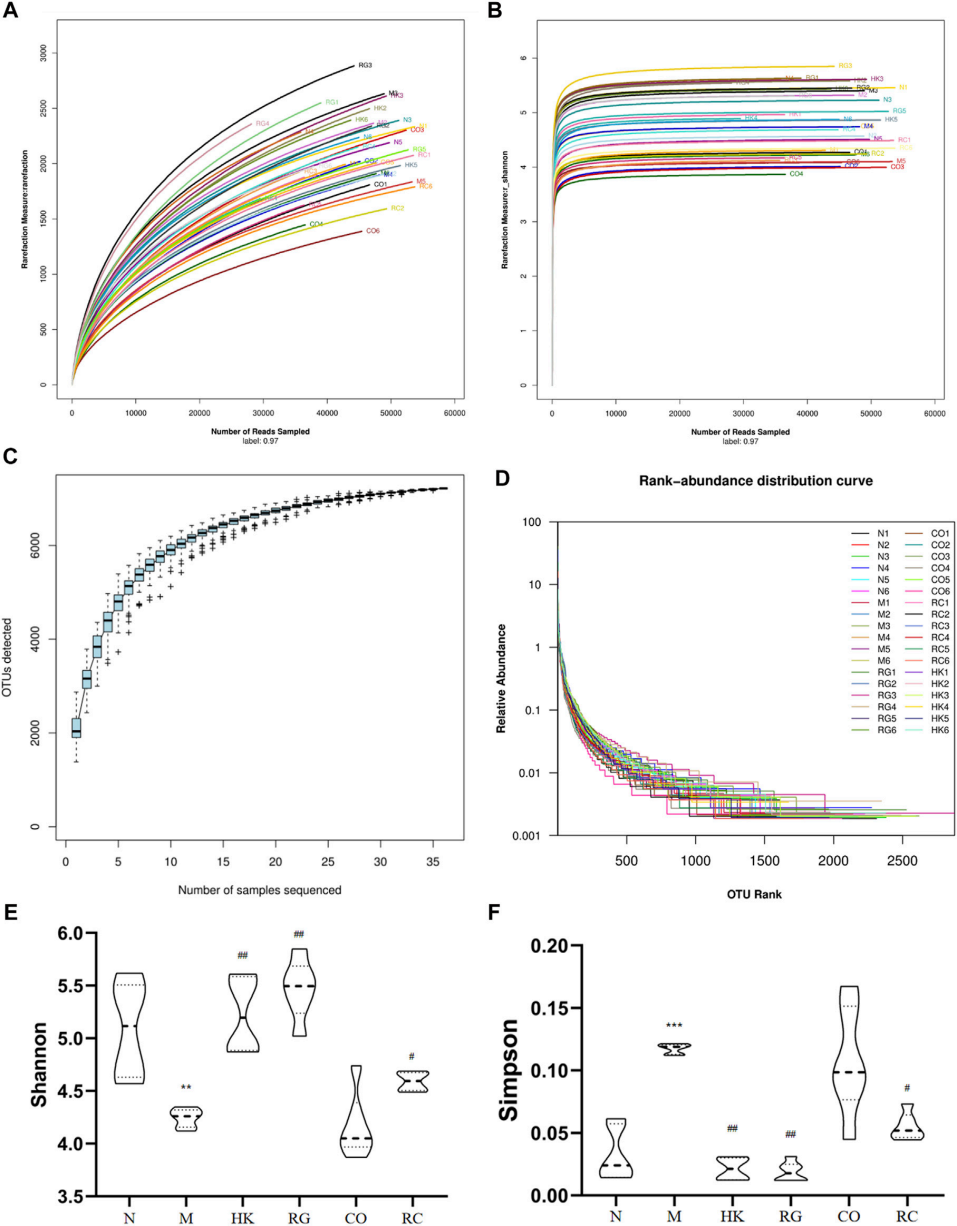
Changes in the microbial community diversity and structure. **(A)** Rarefaction curves, **(B)** Shannon–Wiener curves, **(C)** species accumulation curves, **(D)** rank-abundance distribution curve, **(E)** Shannon–Wiener curves, and **(F)** Simpson curves. (Data are means ± SD. **p < 0.01 vs*.* normal group; ^##^ *p <* 0.01 and ^#^ *p <* 0.05 vs*.* model group. *n* = 6).

In the present study, adenine-induced rats had a significantly lower Shannon index (*p* < 0.05) than the normal group, indicating lower microbial community richness, while the HK, RG, CO, and RC groups showed increased abundance (Figure 6). In addition, the Simpson index level was higher in the M group (*p* < 0.05), indicating less diversity of the microbial community (Figure 6). In contrast, microbial diversity was increased in HK-, RG-, and RC-treated rats (Figure 6). In short, compared with HK, RG, and RC treatment, this could significantly reverse the decrease in gut microbiota abundance but had little effect on community diversity. The individual rarefaction curves, Shannon Wiener curves, species accumulation curves, and rank-abundance distribution curve of samples were close to the saturation plateau (Figure 6), resulting in high sampling coverage and an adequate diversity of species in all samples.

**FIGURE 7:**
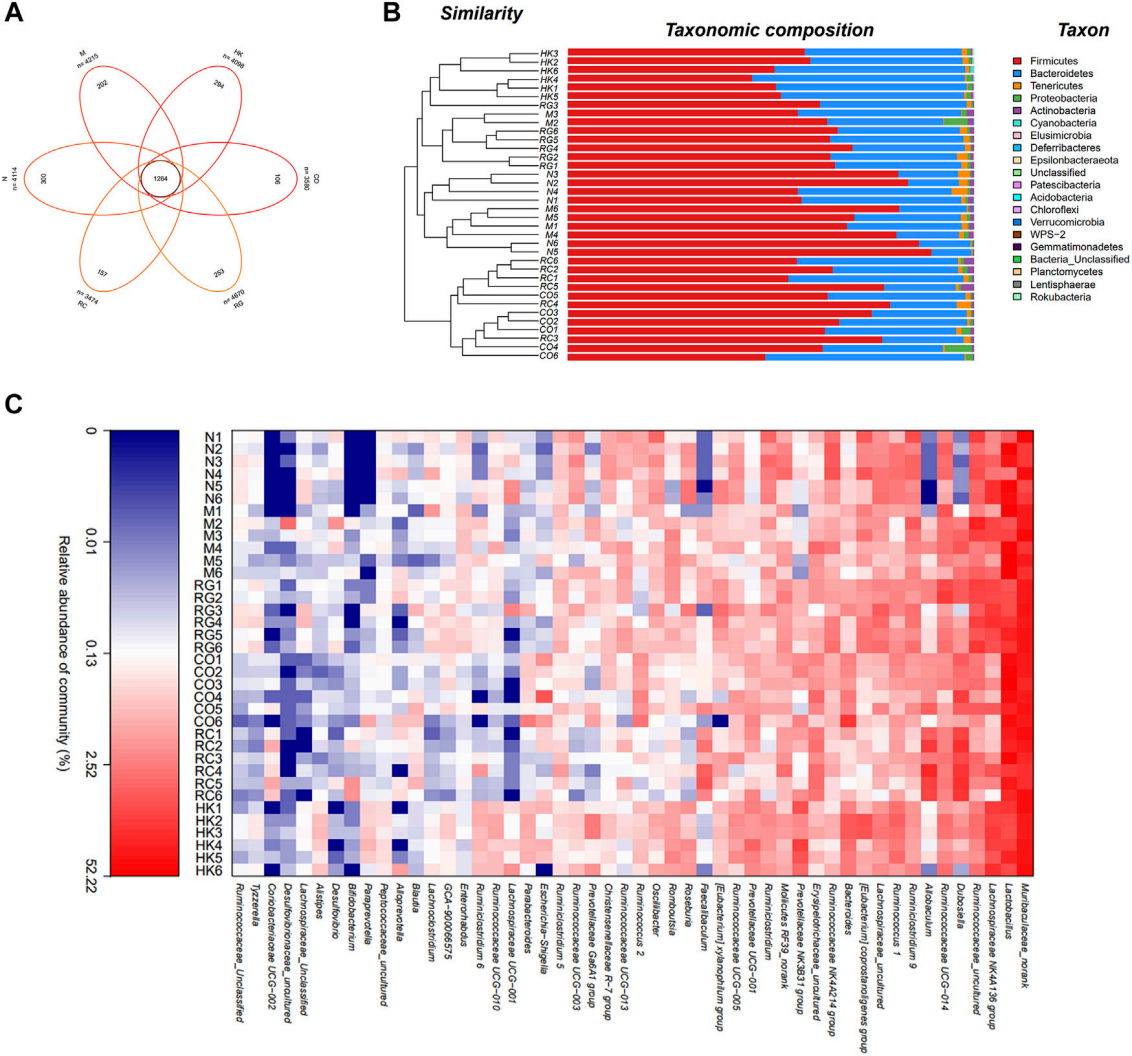
Intestinal bacterial composition of CKD rats. **(A)** OTU Venn analysis, **(B)** microbial community barplot with cluster tree, and **(C)** microbial community heatmap analysis.

Venn diagrams were used to determine which OTUs were unique to different treatment groups. Figure 7 shows that there were 202 and 300 unique OTUs in the model and control mice, respectively. On the contrary, in the treatment group, 106, 253, and 157 OTUs were distinct between CO, RG, and RC groups, respectively.

Furthermore, the histograms of the communities showed little difference in the overall community structure at the phylum level. The main phylum levels in each group were Firmicutes, Bacteroidetes, Tenericutes, Leptidobacterium, Proteobacteria, and Actinobacteria, of which Firmicutes was the most important phylum in the sample intestinal bacteria, followed by Bacteroidetes phylum. Compared with normal rats, the abundance of Firmicutes in CKD rats was relatively increased, while that of the Bacteroidetes was decreased. Studies have shown that the imbalance between the Firmicutes and Bacteroidetes ratios was largely related to energy metabolism (Lin et al., 2018). A significant increase in the F/B ratios was seen in CKD rats in comparison with the normal group, which then decreased after treatment with RC. Heatmap analysis revealed that the predominant bacteria in the RC and N groups were relatively similar compared to the M group, suggesting that RC could regulate the gut bacteria of key phylotypes in adenine-induced CKD rats.

A distance matrix was calculated from beta diversity analysis, which was used to conduct hierarchical clustering of samples to determine the similarity in species composition between the samples. The results showed that biological replicates from the same experimental group (N, M, RG, CO, and RC) clustered together. This indicated that the intestinal flora in different treatment group results in a distinct and reproducible compositional expression (Figure 7).

### 3.7 Comparison of the gut bacterial community among different treatment groups

**FIGURE 8:**
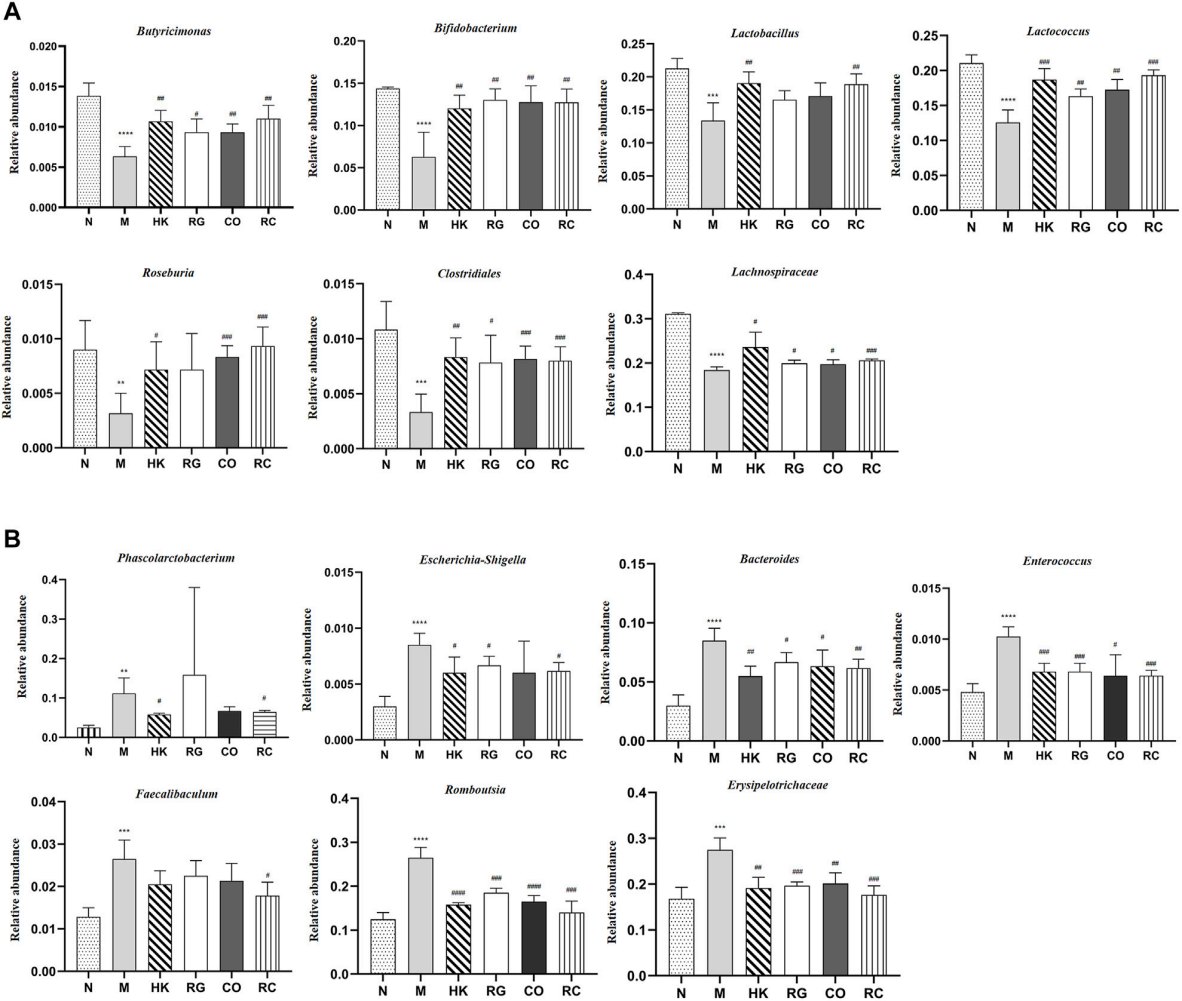
**(A)** Relative abundance of predominant bacteria. **(B)** Relative abundance of pernicious bacteria. (Data are means ± SD. ****p < 0.0001, ***p < 0.001, **p < 0.01, and *p < 0.05 vs*.* normal group; ^###^ *p <* 0.001, ^##^ *p <* 0.01, and ^#^ *p <* 0.05 vs*.* model group. *n* = 6).

In this study, different treatment groups were found to exhibit significant differences in the diversity and richness of intestinal flora. At the genus level, there were significant differences in the number of 42 species that were found between adenine-induced CKD rats and the normal group, of which 12 species were classified. Compared with the normal group, the relative abundance of *Bacteroides*, *Phascolarctobacterium*, *Escherichia*, *Shigella*, *Enterococcus*, *Faecalibaculum*, *Romboutsia*, and Erysipelotrichaceae in adenine-induced CKD rats was increased significantly (*p* < 0.05 and *p* < 0.01; Figure 8B), but the abundance of *Butyricimonas*, *Bifidobacterium*, *Lactobacillus*, *Lactococcus*, *Roseburia*, Clostridiales, and Lachnospiraceae was decreased significantly (*p* < 0.05 and *p* < 0.01; Figure 8A). After RC treatment, the lowered relative abundance of *Candidatus Soleaferrea*, *Globicatella* Lachnospiraceae, *Butyricimonas*, and *Ruminococcus* 1 in adenine-induced CKD rats was increased significantly (*p* < 0.05 and *p* < 0.01; Figure 8A). In addition, the relative abundance of *Bacteroides*, *Escherichia*, *Shigella*, *Enterococcus*, *Faecalibaculum*, *Romboutsia*, and Erysipelotrichaceae was decreased significantly by RG, CO, and RC vs. the model group (*p* < 0.05 and *p* < 0.01 Figure 8B).

**FIGURE 9:**
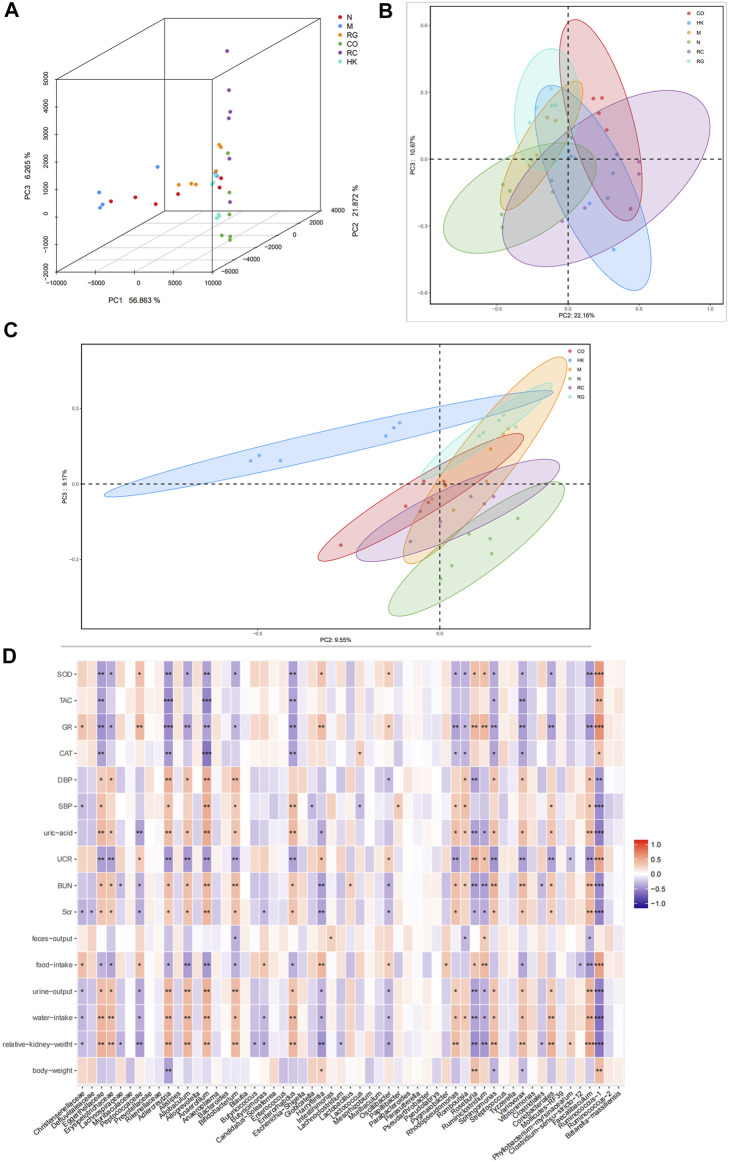
**(A)** Multiple samples of 3D PCA. **(B–C)** PCoA based on weight_unifrac and unweight_unifrac distance algorithms. **(D)** Relationship between SOD, TAC, GR, and CAT and the aforementioned intestinal microbiota.

According to a PCA and a PCoA based on a distance matrix (Bray–Curtis algorithm), there was a clear separation of gut microbiome profiles between normal rats and model rats in terms of beta diversity. In Figure 9, it is clear that the plot for the RC group was closer to that of the normal group, suggesting that the RC group had higher similarities among bacterial members with N group rats.

PCoA and 3D PCA confirmed that the N, M, and RC group rats clustered clearly within their own groups, according to the hierarchical clustering analysis based on the unweighted pair group method with arithmetic mean (UPGMA) (Figure 9). According to the distance matrix (Bray–Curtis algorithm) obtained from beta diversity analysis, a hierarchical cluster analysis was performed with the R language tool to determine the similarity of species composition between samples through UPGMA. Closer samples had shorter branches, indicating a similar species composition. As a result of these findings, it is possible that the modulation of the gut microbiota structure may contribute to the amelioration of RG, CO, and RC.

### 3.8 Correlation between gut bacteria and biochemical parameters

In order to investigate the potential association between gut microbiota alteration and host phenotypes, with Spearman correlation analysis, we evaluated the relationship between relative abundances of the most abundant genera and the biochemical parameters. The results indicated that the levels of uric acid, BUN, SCr, urine output, water intake, and relative kidney weight correlated positively with Eggerthellaceae, Erysipelotrichaceae, *Adlercreutzia*, *Alloprevotella*, *Allobaculum*, *Anaerofilum*, *Enterococcus*, *Rhodopseudomonas*, *Faecalibaculum*, and *Sphingomonas*. On the contrary, the levels of SOD, TAC, GR, and CAT appeared to be negatively correlated with the aforementioned gut microbiota (Figure 9).

## 4 Discussion

Chronic kidney disease is a clinical syndrome with slow development and gradual deterioration of renal function. Its pathogenesis is complex, its course is long, its condition is serious, and often it continues to progress; eventually, it develops into end-stage renal failure, which seriously endangers human health and life. Delaying or stopping the progression of CKD has become an important challenge for clinicians and health authorities around the world. At present, modern medicine still lacks ideal treatment methods for this disease, while traditional Chinese medicine has accumulated rich clinical practice experience in the treatment of chronic kidney disease, which can effectively improve renal function and delay renal failure, with unique advantages. In view of the increasing amount of evidence to suggest TCMs/TCM formulas may act as prebiotics to modulate gut microbiota composition and metabolic phenotype, in recent years, it has become apparent that TCM formulas may be used as prebiotics to modify gut microbiota structure and metabolic phenotypes in the host, potentially providing new drug leads (Chang et al., 2017; Yu et al., 2017).

The intestinal tract of the human body is symbiotic with a large number of microorganisms, which constitute an extremely complex micro-ecosystem and maintain a relatively stable balance. The epidemic affects the immune-inflammatory anti-inflammatory axis (Aronov et al., 2011; Holmes et al., 2012; Nicholson et al., 2012; Valentina and Fredrik, 2012), which plays an important role in maintaining human health. In 2011, Meijiers et al. (Aronov et al., 2011) proposed the “gut-kidney Axis” theory of the progression of CKD drying and intervention. Intestinal barrier injury, resulting in intestinal bacteria and endotoxin translocation, is an important mechanism of kidney injury in the “gut-kidney” axis. Injury to the intestinal mucosal barrier, resulting in the translocation of intestinal bacteria, penetrating into the mucosa of the anesthetic tract, causing the immune system to abnormally regulate the intestinal bacteria or its metabolites, and also showing some normal damage to the bacterial flora, does not cause an immune response, leading to inflammation. As the wall of the intestinal mucosal barrier is constructed, the intestinal mucosal epithelium’s integrity and regeneration capacity play a crucial role. The connections between intestinal epithelial cells, from the top to the basement membrane, are tight junctions, adhesion junctions, desmosomes, and gap junctions. Among them, tight junctions are composed of structural proteins such as JAM, occludin-1, ZOs (zonula occludens), claudin-1, and various connexin molecules. It is the most important structure that constitutes the mechanical barrier of the intestinal mucosa. It plays an important role in the media, and the tight junction adhesion complex controls the integrity of the epithelial barrier.

In this study, the CKD rat model induced by adenine was successfully established, and the possible mechanisms of the *Rehmannia glutinosa* Libosch and *Cornus officinalis* Sieb herb couple on improving chronic kidney disease were explored by regulating the intestinal flora and enhancing the intestinal barrier.

Our sequencing analysis of 16S rDNA in microbial samples revealed that chronic kidney disease, induced by adenine, could alter the composition of gut bacteria significantly. Rats with CKD showed significant decreases in alpha diversity in comparison to normal rats; however, RG and RC could increase the alpha diversity of the gut bacteria in CKD rats. Meanwhile, this study clearly demonstrated that the gut dysbiosis of adenine-induced CKD rats was characterized by the intestinal flora abundant in *Bacteroides*, *Phascolarctobacterium*, *Escherichia*, *Shigella*, *Enterococcus*, *Faecalibaculum*, *Romboutsia*, and Erysipelotrichaceae, while less in *Butyricimonas*, *Bifidobacterium*, *Lactobacillus*, *Lactococcus*, *Roseburia*, Clostridiales, and Lachnospiraceae. Previous animal experiments and human studies have consistently shown that gut dysbiosis in CKD patients is mainly attributable to the reduction of bifidobacteria, and CKD progression is closely related to a lower abundance of *Roseburia* (Liu et al., 2018). A clinical study was conducted in patients with ESRD to investigate the role of gut microbiota in the generation of uremic toxins associated with negative outcomes. In their study, six taxa (*Enterococcus*, *Akkermansia*, *Dialister*, *Ruminococcus*, *Bacteroides*, and *Blautia*) correlated with increased uremic toxins and would need further investigation as microbial targets to lower uremic toxin concentrations to improve outcomes in patients with CKD (Joossens et al., 2019). As uremic toxins and urea enter the GI lumen, they imposed a selective pressure that encourages the growth of bacteria that produce urease, indole, and p-cresol-forming enzymes, causing a vicious cycle of inflammation and oxidative stress at the renal level (Vaziri et al., 2013). In addition, the profile of gut dysbiosis in the CKD rats of the present study, especially the reduction of *Bifidobacterium* and *Roseburia*, was significantly associated with inflammatory factors and brain phenotypes. The beneficial health effects of *Bifidobacterium* and *Roseburia* have been shown to be probiotics that enhance cognitive performance by metabolizing the host’s glutamate (Jiang et al., 2017; Zheng et al., 2020). SCFAs are mainly produced by bacterial metabolism and are an important energy source for colonic epithelial cells, which can enhance the integrity of the epithelial barrier and activate the gastrointestinal immune response, especially butyrate. It has been reported that *Roseburia* and *Butyricicoccus*, the major butyrate-producing bacteria, are typically found in low levels in the human gut with inflammatory diseases (Jiang et al., 2017; Garg and Ooi, 2017; Tao et al., 2017). The findings showed that treatment with the *Rehmannia glutinosa* Libosch and *Cornus officinalis* Sieb herb couple could maintain/shift the overall structure of adenine-induced CKD rats’ gut microbiota toward that of normal control rats.

Another important consideration is identifying the key microorganisms responsible for RC intervention that prevents CKD. After analysis by LEfSe and Spearman’s correlations, the results showed that the levels of uric acid, BUN, SCr, urine output, water intake, and relative kidney weight presented a notable positive correlation with Eggerthellaceae, Erysipelotrichaceae, *Adlercreutzia*, *Alloprevotella*, *Allobaculum*, *Anaerofilum*, *Enterococcus*, *Rhodopseudomonas*, *Faecalibaculum*, and *Sphingomonas*. On the contrary, the levels of SOD, TAC, GR, and CAT appeared to have a negative correlation with the aforementioned gut microbiota. Hemodialysis patients have been reported to benefit from *Anaerofilum* as it regulates the absorption of phosphate in the intestine by phosphorus metabolism (Miao et al., 2018). Eggerthellaceae is another family implicated in CKD and hemodialysis progression by its higher abundance in CKD patients than in controls (Lun et al., 2019).

It is now widely recognized that an undamaged gut barrier has an important function in preventing intestinal bacteria, toxins, or allergens from entering the human body through the gut. In this work, we assessed intestinal permeability using an *ex vivo* Ussing chamber. Decreased TER and increased FD4 flux have been reported to signal the impaired gut barrier (Jiao et al., 2015). In this experiment, the *Rehmannia glutinosa* Libosch and *Cornus officinalis* Sieb herb couple extract increased the transepithelial electrical resistance in colon tissue and decreased fluorescein isothiocyanate–dextran 4 kDa flux in colon tissue of the adenine-induced CKD rats. At the same time, the RG and CO groups had no effect in the aforementioned parameters. This finding indicates that the *Rehmannia glutinosa* Libosch and *Cornus officinalis* Sieb herb couple extract might benefit gut barrier function.

Evidence is emerging that intestinal barrier dysfunction contributes to the pathogenesis of systemic inflammation in CKD patients and animals. Furthermore, to elucidate the molecular mechanisms behind the effects of the *Rehmannia glutinosa* Libosch and *Cornus officinalis* Sieb herb couple extract on gut barrier function, we examined whether RC supplementation influenced the tight junction protein expression in rats with CKD. In rats’ intestines, occluden-1, claudin-1, and ZO-1, which were the cell connection points between cells, formed the tight junction (Jiao et al., 2017). In this study, the protein expressions of occludin-1, claudin-1, and ZO-1 were decreased significantly in chronic kidney disease rats with the induction of adenine (Lin et al., 2018; Liu et al., 2018). With the treatment of RG, CO, and RC, the intestinal barrier was repaired due to the upregulated expressions of the abovementioned proteins in CKD rats (Figure 5). Meanwhile, the mRNA expressions of such tight junction-related genes, which were tested by qRT-PCR, were also significantly decreased in CKD rats. After RC treatment, the corresponding mRNA expression was significantly increased. This corresponded to the results from the Ussing chamber experiment. The gut barrier, consisting of enterocytes and an extracellular mucin layer, protects the body’s internal environment from direct contact with the microbiota. At the same time, the composition of mucins, the characteristics of tight junctions, and a large number of active substance transport systems, allow for complex bidirectional metabolic and immune crosstalk. Loss or damage of kidney function directly interferes with the function of the intestinal barrier, resulting in decreased intestinal barrier effectiveness and increased exposure to bacterial products. CKD also interacts with active transport mechanisms in complex ways, thereby altering the homeostasis of many solutes. The specific mechanism of action will continue to be further studied in the later stage. Together, our results indicate that RC treatment modulates gut microbiota and protects intestinal barrier integrity in CKD rats, possibly through upregulation of tight junction genes, thereby alleviating the occurrence and development of chronic kidney disease.

## 5 Conclusion

In conclusion, the purpose of this study was to investigate the effects of the *Rehmannia glutinosa* Libosch and *Cornus officinali*s Sieb herb couple on adenine-induced CKD rats by modulating the intestinal microbiota and enhancing the intestinal barrier function. Our data indicate that RC strengthens the intestinal barrier and modulates gut microbiota in rats treated with adenine-induced CKD. This project revealed the compatibility mechanism of *Rehmannia glutinosa* and *Cornus officinalis* in regulating intestinal micro-ecology and barrier function to intervene in CKD and provided the basis and ideas for the clinical application of RC and the development of innovative drugs for CKD.

However, looking to the future, aiming at the two core links of intestinal flora imbalance and intestinal barrier damage in the “intestinal-renal” axis, they may be the effective targets for clinical treatment of CKD.

Under normal physiological conditions, the intestinal flora maintains a dynamic balance in the human body, digests and absorbs dietary components to provide energy for the body, at the same time, promotes the production of beneficial metabolites such as short-chain fatty acids, protects the intestinal mucosal barrier function, and maintains the homeostasis of the intestinal lumen so that the body can carry out normal physiological activities. With the occurrence and development of chronic kidney disease, drug therapy intervention and a large amount of urea decomposed in the intestinal lumen affect the changes of the intestinal lumen environment and are biased toward the growth and colonization of pathogenic bacteria. The disorder between beneficial bacteria and pathogenic bacteria leads to the imbalance between saccharification fermentation and protein fermentation, resulting in the reduction of short-chain fatty acids and the increase of urinary toxins, breaking the intestinal barrier, causing the translocation of bacteria and their metabolites, resulting in abnormal insulin disturbance of sugar and homeostasis, and accelerating disease progression.

The use of prebiotics, probiotics, and symbiotic supplements has emerged as a potential therapeutic intervention to restore intestinal flora imbalance. The experimental evidence so far is promising, but we still need more evidence from further clinical research to confirm the efficacy and safety of using these “organisms” as a treatment tool for CKD.

## Data Availability

The original contributions presented in the study are included in the article/Supplementary Material; further inquiries can be directed to the corresponding author.

## Abbreviations

4 kDa FITC-dextran; FD4, the flux of 4-kilodalton fluorescein isothiocyanate–dextran; CAT, renal catalase; CKD, chronic kidney disease; CO, *Cornus officinalis* Sieb; DBP, diastolic blood pressure; ELISA, enzyme-linked immunosorbent assay; GR, glutathione reductase; OTU, operational taxonomic unit; PMSF, phenylmethanesulfonyl fluoride; RC, *Rehmannia glutinosa*—*Cornus officinalis* herb couple; RG, *Rehmannia glutinosa* Libosch; SBP, systolic blood pressure; SCFAs, short-chain fatty acids; SDS-PAGE, sodium dodecyl sulfate–polyacrylamide gel electrophoresis; SOD, superoxide dismutase; TAC, total antioxidant capacity; TCM, traditional Chinese medicine; TER, transepithelial electrical resistance; TJ, tight junction; TLR4, Toll-like receptor 4; UP, urinary protein; ZO, zonula occludens.
