# Supplementary material for: Preventive effects of the Rehmannia glutinosa Libosch and Cornus officinalis Sieb herb couple on chronic kidney disease rats via modulating the intestinal microbiota and enhancing the intestinal barrier
Source: Front Pharmacol. 2022 Sep 8;13:942032. doi: 10.3389/fphar.2022.942032 (PMC9495080; doi:10.3389/fphar.2022.942032)
Supplement: Supplementary file 1 [file Table1.docx]

| **sample** | **primer** | CT | | | | | **SD** | CT average | **ΔCT** | **claudin-1/β-actin 2**-**^△^**CT |
| --- | --- | --- | --- | --- | --- | --- | --- | --- | --- | --- |
| **N** | β-actin | 25.711 | | 25.574 | | 25.681 | 0.072 | 25.655 | 0.000 | 1.00000 |
|  | claudin-1 | 26.119 | | 30.285 | | 30.828 | 2.576 | 28.474 | 2.818 | 0.14177 |
| **sample** | **primer** | CT | | | | | **SD** | CT average | **ΔCT** | **claudin-1/β-actin 2**-**^△^**CT |
| **N** | β-actin | 25.735 | | 25.933 | | 25.836 | 0.099 | 25.835 | 0.000 | 1.00000 |
|  | claudin-1 | 27.917 | | 30.619 | | 30.416 | 1.505 | 29.167 | 3.332 | 0.09930 |
| **sample** | **primer** | CT | | | | | **SD** | CT average | **ΔCT** | **claudin-1/β-actin 2**-**^△^**CT |
| **N** | β-actin | 25.979 | | 25.850 | | 25.897 | 0.065 | 25.909 | 0.000 | 1.00000 |
|  | claudin-1 | 28.491 | | 30.500 | | 30.853 | 1.274 | 29.672 | 3.763 | 0.07365 |
| **sample** | **primer** | CT | | | | | **SD** | CT average | **ΔCT** | **claudin-1/β-actin 2**-**^△^**CT |
| **N** | β-actin | 18.953 | | 19.003 | | 18.984 | 0.025 | 18.980 | 0.000 | 1.00000 |
|  | claudin-1 | 23.916 | | 27.906 | | 27.026 | 2.096 | 25.471 | 6.491 | 0.01111 |
| **sample** | **primer** | CT | | | | | **SD** | CT average | **ΔCT** | **claudin-1/β-actin 2**-**^△^**CT |
| **N** | β-actin | 23.796 | | 23.964 | | 23.877 | 0.084 | 23.879 | 0.000 | 1.00000 |
|  | claudin-1 | 29.680 | | 30.145 | | 30.665 | 0.492 | 30.172 | 6.293 | 0.01275 |
| **sample** | **primer** | CT | | | | | **SD** | CT average | **ΔCT** | **claudin-1/β-actin 2**-**^△^**CT |
| **N** | β-actin | 22.013 | | 22.110 | | 22.271 | 0.130 | 22.131 | 0.000 | 1.00000 |
|  | claudin-1 | 27.409 | | 29.634 | | 30.053 | 1.421 | 28.731 | 6.600 | 0.01031 |
| **sample** | **primer** | CT | | | | | **SD** | CT average | **ΔCT** | **claudin-1/β-actin 2**-**^△^**CT |
| **M** | β-actin | 25.048 | | 25.074 | | 24.949 | 0.066 | 25.024 | 0.000 | 1.00000 |
|  | claudin-1 | 29.988 | | 30.452 | | 30.226 | 0.232 | 30.107 | 5.083 | 0.02950 |
| **sample** | **primer** | CT | | | | | **SD** | CT average | **ΔCT** | **claudin-1/β-actin 2**-**^△^**CT |
| **M** | β-actin | 24.050 | | 24.065 | | 24.045 | 0.011 | 24.053 | 0.000 | 1.00000 |
|  | claudin-1 | 29.800 | | 29.950 | | 29.927 | 0.081 | 29.863 | 5.810 | 0.01782 |
| **sample** | **primer** | CT | | | | | **SD** | CT average | **ΔCT** | **claudin-1/β-actin 2**-**^△^**CT |
| **M** | β-actin | 22.913 | | 22.943 | | 22.941 | 0.017 | 22.932 | 0.000 | 1.00000 |
|  | claudin-1 | 30.382 | | 29.636 | | 29.868 | 0.382 | 30.125 | 7.193 | 0.00683 |
| **sample** | **primer** | CT | | | | | **SD** | CT average | **ΔCT** | **claudin-1/β-actin 2**-**^△^**CT |
| **M** | β-actin | 19.862 | | 19.805 | | 19.908 | 0.051 | 19.858 | 0.000 | 1.00000 |
|  | claudin-1 | 29.942 | | 29.794 | | 29.681 | 0.131 | 29.812 | 9.953 | 0.00101 |
| **sample** | **primer** | CT | | | | | **SD** | CT average | **ΔCT** | **claudin-1/β-actin 2**-**^△^**CT |
| **M** | β-actin | 20.934 | | 20.762 | | 20.456 | 0.242 | 20.718 | 0.000 | 1.00000 |
|  | claudin-1 | 29.420 | | 27.893 | | 29.159 | 0.817 | 29.290 | 8.572 | 0.00263 |
| **sample** | **primer** | CT | | | | | **SD** | CT average | **ΔCT** | **claudin-1/β-actin 2**-**^△^**CT |
| **M** | β-actin | 17.965 | | 17.950 | | 17.940 | 0.012 | 17.952 | 0.000 | 1.00000 |
|  | claudin-1 | 28.022 | | 28.460 | | 28.590 | 0.297 | 28.306 | 10.355 | 0.00076 |
| **sample** | **primer** | CT | | | | | **SD** | CT average | **ΔCT** | **claudin-1/β-actin 2**-**^△^**CT |
| **HK** | β-actin | 25.635 | | 25.569 | | 25.526 | 0.055 | 25.577 | 0.000 | 1.00000 |
|  | claudin-1 | 29.676 | | 30.366 | | 30.264 | 0.372 | 29.970 | 4.393 | 0.04758 |
| **sample** | **primer** | CT | | | | | **SD** | CT average | **ΔCT** | **claudin-1/β-actin 2**-**^△^**CT |
| **HK** | β-actin | 25.069 | | 24.991 | | 25.065 | 0.044 | 25.042 | 0.000 | 1.00000 |
|  | claudin-1 | 30.365 | | 30.442 | | 30.323 | 0.060 | 30.344 | 5.303 | 0.02533 |
| **sample** | **primer** | **CT** | | | | | **SD** | **CT average** | **ΔCT** | **claudin-1/β-actin 2**-**^△^**CT |
| **HK** | β-actin | 22.114 | | 22.096 | | 22.119 | 0.012 | 22.110 | 0.000 | 1.00000 |
|  | claudin | 28.045 | | 28.431 | | 28.932 | 0.445 | 28.489 | 6.379 | 0.01202 |
| **sample** | **primer** | **CT** | | | | | **SD** | **CT average** | **ΔCT** | **claudin-1/β-actin 2**-**^△^**CT |
| **HK** | β-actin | 21.935 | | 21.455 | | 21.369 | 0.305 | 21.586 | 0.000 | 1.00000 |
|  | claudin | 30.644 | | 29.558 | | 29.461 | 0.657 | 30.052 | 8.466 | 0.00283 |
| **sample** | **primer** | **CT** | | | | | **SD** | **CT average** | **ΔCT** | **claudin-1/β-actin 2**-**^△^**CT |
| **HK** | β-actin | 19.738 | | 19.916 | | 19.617 | 0.150 | 19.757 | 0.000 | 1.00000 |
|  | claudin | 26.634 | | 26.197 | | 26.688 | 0.269 | 26.661 | 6.904 | 0.00835 |
| **sample** | **primer** | **CT** | | | | | **SD** | **CT average** | **ΔCT** | **claudin-1/β-actin 2**-**^△^**CT |
| **HK** | β-actin | 21.843 | | 21.845 | | 21.953 | 0.063 | 21.880 | 0.000 | 1.00000 |
|  | claudin | 31.333 | | 30.999 | | 29.816 | 0.797 | 30.575 | 8.694 | 0.00241 |
| **sample** | **primer** | CT | | | | | **SD** | CT average | **ΔCT** | **claudin-1/β-actin 2**-**^△^**CT |
| **RG** | β-actin | 23.737 | | 24.048 | | 23.938 | 0.157 | 23.907 | 0.000 | 1.00000 |
|  | claudin-1 | 29.892 | | 29.446 | | 29.484 | 0.248 | 29.688 | 5.781 | 0.01819 |
| **sample** | **primer** | CT | | | | | **SD** | CT average | **ΔCT** | **claudin-1/β-actin 2**-**^△^**CT |
| **RG** | β-actin | 23.223 | | 23.196 | | 23.311 | 0.060 | 23.243 | 0.000 | 1.00000 |
|  | claudin-1 | 29.278 | | 29.833 | | 29.399 | 0.292 | 29.338 | 6.095 | 0.01463 |
| **sample** | **primer** | CT | | | | | **SD** | CT average | **ΔCT** | **claudin-1/β-actin 2**-**^△^**CT |
| **RG** | β-actin | 22.312 | | 22.129 | | 22.239 | 0.092 | 22.227 | 0.000 | 1.00000 |
|  | claudin-1 | 29.747 | | 29.781 | | 29.936 | 0.101 | 29.842 | 7.615 | 0.00510 |
| **sample** | **primer** | CT | | | | | **SD** | CT average | **ΔCT** | **claudin-1/β-actin 2**-**^△^**CT |
| **RG** | β-actin | 21.012 | | 20.889 | | 21.094 | 0.103 | 20.998 | 0.000 | 1.00000 |
|  | claudin-1 | 29.670 | | 29.901 | | 29.594 | 0.160 | 29.632 | 8.634 | 0.00252 |
| **sample** | **primer** | CT | | | | | **SD** | CT average | **ΔCT** | **claudin-1/β-actin 2**-**^△^**CT |
| **RG** | β-actin | 22.842 | | 23.335 | | 22.694 | 0.336 | 22.957 | 0.000 | 1.00000 |
|  | claudin-1 | 30.188 | | 31.825 | | 31.720 | 0.916 | 30.954 | 7.997 | 0.00392 |
| **sample** | **primer** | CT | | | | | **SD** | CT average | **ΔCT** | **claudin-1/β-actin 2**-**^△^**CT |
| **RG** | β-actin | 19.765 | | 20.288 | | 19.809 | 0.290 | 19.954 | 0.000 | 1.00000 |
|  | claudin-1 | 31.064 | | 31.292 | | 30.961 | 0.169 | 31.013 | 11.059 | 0.00047 |
| **sample** | **primer** | CT | | | | | **SD** | CT average | **ΔCT** | **claudin-1/β-actin 2**-**^△^**CT |
| **CO** | β-actin | 22.643 | | 22.613 | | 22.556 | 0.044 | 22.604 | 0.000 | 1.00000 |
|  | claudin-1 | 30.956 | | 30.941 | | 31.473 | 0.303 | 31.215 | 8.611 | 0.00256 |
| **sample** | **primer** | CT | | | | | **SD** | CT average | **ΔCT** | **claudin-1/β-actin 2**-**^△^**CT |
| **CO** | β-actin | 18.911 | | 18.795 | | 18.902 | 0.065 | 18.869 | 0.000 | 1.00000 |
|  | claudin-1 | 27.894 | | 27.899 | | 27.836 | 0.035 | 27.865 | 8.996 | 0.00196 |
| **sample** | **primer** | CT | | | | | **SD** | CT average | **ΔCT** | **claudin-1/β-actin 2**-**^△^**CT |
| **CO** | β-actin | 20.935 | | 20.164 | | 20.558 | 0.386 | 20.552 | 0.000 | 1.00000 |
|  | claudin-1 | 29.975 | | 30.161 | | 29.907 | 0.131 | 29.941 | 9.389 | 0.00149 |
| **sample** | **primer** | CT | | | | | **SD** | CT average | **ΔCT** | **claudin-1/β-actin 2**-**^△^**CT |
| **CO** | β-actin | 19.936 | | 19.901 | | 19.858 | 0.039 | 19.898 | 0.000 | 1.00000 |
|  | claudin-1 | 31.009 | | 31.122 | | 29.859 | 0.699 | 30.434 | 10.536 | 0.00067 |
| **sample** | **primer** | CT | | | | | **SD** | CT average | **ΔCT** | **claudin-1/β-actin 2**-**^△^**CT |
| **CO** | β-actin | 20.225 | | 20.152 | | 20.565 | 0.221 | 20.314 | 0.000 | 1.00000 |
|  | claudin-1 | 30.943 | | 31.054 | | 30.411 | 0.344 | 30.677 | 10.363 | 0.00076 |
| **sample** | **primer** | CT | | | | | **SD** | CT average | **ΔCT** | **claudin-1/β-actin 2**-**^△^**CT |
| **CO** | β-actin | 20.652 | | 20.420 | | 20.511 | 0.117 | 20.528 | 0.000 | 1.00000 |
|  | claudin-1 | 31.104 | | 30.828 | | 31.263 | 0.220 | 31.183 | 10.656 | 0.00062 |
| **sample** | **primer** | CT | | | | | **SD** | CT average | **ΔCT** | **claudin-1/β-actin 2**-**^△^**CT |
| **RC** | β-actin | 23.522 | | 23.566 | | 23.473 | 0.047 | 23.520 | 0.000 | 1.00000 |
|  | claudin-1 | 29.049 | | 29.183 | | 29.911 | 0.464 | 29.480 | 5.960 | 0.01607 |
| **sample** | **primer** | CT | | | | | **SD** | CT average | **ΔCT** | **claudin-1/β-actin 2**-**^△^**CT |
| **RC** | β-actin | 25.320 | | 25.476 | | 25.231 | 0.124 | 25.342 | 0.000 | 1.00000 |
|  | claudin-1 | 29.443 | | 29.607 | | 29.866 | 0.213 | 29.655 | 4.312 | 0.05034 |
| **sample** | **primer** | CT | | | | | **SD** | CT average | **ΔCT** | **claudin-1/β-actin 2**-**^△^**CT |
| **RC** | β-actin | 24.496 | | 24.936 | | 24.574 | 0.234 | 24.669 | 0.000 | 1.00000 |
|  | claudin-1 | 29.717 | | 29.310 | | 29.900 | 0.302 | 29.809 | 5.140 | 0.02836 |
| **sample** | **primer** | CT | | | | | **SD** | CT average | **ΔCT** | **claudin-1/β-actin 2**-**^△^**CT |
| **RC** | β-actin | 21.437 | | 21.466 | | 21.531 | 0.048 | 21.478 | 0.000 | 1.00000 |
|  | claudin-1 | 28.887 | | 29.355 | | 28.914 | 0.263 | 28.901 | 7.422 | 0.00583 |
| **sample** | **primer** | CT | | | | | **SD** | CT average | **ΔCT** | **claudin-1/β-actin 2**-**^△^**CT |
| **RC** | β-actin | 21.937 | | 21.968 | | 21.971 | 0.019 | 21.959 | 0.000 | 1.00000 |
|  | claudin-1 | 28.450 | | 29.481 | | 28.952 | 0.516 | 28.701 | 6.742 | 0.00934 |
| **sample** | **primer** | CT | | | | | **SD** | CT average | **ΔCT** | **claudin-1/β-actin 2**-**^△^**CT |
| **RC** | β-actin | 21.585 | | 21.706 | | 21.652 | 0.061 | 21.648 | 0.000 | 1.00000 |
|  | claudin-1 | 31.356 | | 31.754 | | 27.725 | 2.220 | 29.541 | 7.893 | 0.00421 |
| **sample** | **primer** | CT | | | | | **SD** | CT average | **ΔCT** | **occludin-1/β-actin 2**-**^△^**CT |
| **N** | β-actin | 25.979 | 25.850 | | 25.897 | | 0.065 | 25.909 | 0.000 | 1.00000 |
|  | occludin-1 | 29.408 | 29.920 | | 29.624 | | 0.257 | 29.651 | 3.742 | 0.07473 |
| **sample** | **primer** | **CT** | | | | | **SD** | **CT average** | **ΔCT** | **occludin-1/β-actin 2**-**^△^**CT |
| **N** | β-actin | 25.711 | | 25.574 | | 25.681 | 0.072 | 25.655 | 0.000 | 1.00000 |
|  | occludin-1 | 28.924 | | 28.987 | | 28.867 | 0.155 | 28.926 | 3.271 | 0.10360 |
| **sample** | **primer** | **CT** | | | | | **SD** | **CT average** | **ΔCT** | **occludin-1/β-actin 2**-**^△^**CT |
| **N** | β-actin | 25.735 | | 25.933 | | 25.836 | 0.099 | 25.835 | 0.000 | 1.00000 |
|  | occludin-1 | 29.659 | | 29.929 | | 29.924 | 0.155 | 29.837 | 4.002 | 0.06239 |
| **sample** | **primer** | **CT** | | | | | **SD** | **CT average** | **ΔCT** | **occludin-1/β-actin 2**-**^△^**CT |
| **N** | β-actin | 23.796 | | 23.964 | | 23.877 | 0.084 | 23.879 | 0.000 | 1.00000 |
|  | occludin-1 | 27.738 | | 28.034 | | 30.144 | 1.312 | 28.639 | 4.760 | 0.03691 |
| **sample** | **primer** | **CT** | | | | | **SD** | **CT average** | **ΔCT** | **occludin-1/β-actin 2**-**^△^**CT |
| **N** | β-actin | 22.013 | | 22.110 | | 22.271 | 0.130 | 22.131 | 0.000 | 1.00000 |
|  | occludin-1 | 28.259 | | 28.201 | | 28.400 | 0.102 | 28.287 | 6.155 | 0.01403 |
| **sample** | **primer** | **CT** | | | | | **SD** | **CT average** | **ΔCT** | **occludin-1/β-actin 2**-**^△^**CT |
| **N** | β-actin | 18.953 | | 19.003 | | 18.984 | 0.025 | 18.980 | 0.000 | 1.00000 |
|  | occludin-1 | 25.792 | | 25.790 | | 25.893 | 0.059 | 25.825 | 6.845 | 0.00870 |
| **sample** | **primer** | **CT** | | | | | **SD** | **CT average** | **ΔCT** | **occludin-1/β-actin 2**-**^△^**CT |
| **M** | β-actin | 24.050 | | 24.065 | | 24.045 | 0.011 | 24.053 | 0.000 | 1.00000 |
|  | occludin-1 | 29.119 | | 28.899 | | 29.213 | 0.161 | 29.077 | 5.024 | 0.03074 |
| **sample** | **primer** | **CT** | | | | | **SD** | **CT average** | **ΔCT** | **occludin-1/β-actin 2**-**^△^**CT |
| **M** | β-actin | 25.048 | | 25.074 | | 24.949 | 0.066 | 25.024 | 0.000 | 1.00000 |
|  | occludin-1 | 29.689 | | 29.659 | | 29.801 | 0.099 | 29.716 | 4.692 | 0.03868 |
| **sample** | **primer** | **CT** | | | | | **SD** | **CT average** | **ΔCT** | **occludin-1/β-actin 2**-**^△^**CT |
| **M** | β-actin | 20.934 | | 20.762 | | 20.456 | 0.242 | 20.718 | 0.000 | 1.00000 |
|  | occludin-1 | 26.927 | | 26.915 | | 26.831 | 0.059 | 26.891 | 6.173 | 0.01385 |
| **sample** | **primer** | **CT** | | | | | **SD** | **CT average** | **ΔCT** | **occludin-1/β-actin 2**-**^△^**CT |
| **M** | β-actin | 19.862 | | 19.805 | | 19.908 | 0.051 | 19.858 | 0.000 | 1.00000 |
|  | occludin-1 | 27.352 | | 26.972 | | 27.025 | 0.206 | 27.116 | 7.258 | 0.00653 |
| **sample** | **primer** | **CT** | | | | | **SD** | **CT average** | **ΔCT** | **occludin-1/β-actin 2**-**^△^**CT |
| **M** | β-actin | 22.913 | | 22.943 | | 22.941 | 0.017 | 22.932 | 0.000 | 1.00000 |
|  | occludin-1 | 29.967 | | 30.550 | | 30.859 | 0.453 | 30.459 | 7.526 | 0.00542 |
| **sample** | **primer** | **CT** | | | | | **SD** | **CT average** | **ΔCT** | **occludin-1/β-actin 2**-**^△^**CT |
| **M** | β-actin | 17.965 | | 17.950 | | 17.940 | 0.012 | 17.952 | 0.000 | 1.00000 |
|  | occludin-1 | 27.331 | | 27.188 | | 27.378 | 0.099 | 27.299 | 9.347 | 0.00154 |
| **sample** | **primer** | **CT** | | | | | **SD** | **CT average** | **ΔCT** | **occludin-1/β-actin 2**-**^△^**CT |
| **HK** | β-actin | 25.069 | | 24.991 | | 25.065 | 0.044 | 25.042 | 0.000 | 1.00000 |
|  | occludin-1 | 29.623 | | 29.713 | | 29.620 | 0.053 | 29.652 | 4.610 | 0.04094 |
| **sample** | **primer** | **CT** | | | | | **SD** | **CT average** | **ΔCT** | **occludin-1/β-actin 2**-**^△^**CT |
| **HK** | β-actin | 21.935 | | 21.455 | | 21.369 | 0.305 | 21.586 | 0.000 | 1.00000 |
|  | occludin-1 | 25.591 | | 25.565 | | 26.129 | 0.203 | 25.762 | 4.176 | 0.05532 |
| **sample** | **primer** | **CT** | | | | | **SD** | **CT average** | **ΔCT** | **occludin-1/β-actin 2**-**^△^**CT |
| **HK** | β-actin | 25.635 | | 25.569 | | 25.526 | 0.055 | 25.577 | 0.000 | 1.00000 |
|  | occludin-1 | 30.644 | | 30.902 | | 30.721 | 0.132 | 30.756 | 5.179 | 0.02760 |
| **sample** | **primer** | **CT** | | | | | **SD** | **CT average** | **ΔCT** | **occludin-1/β-actin 2**-**^△^**CT |
| **HK** | β-actin | 19.738 | | 19.916 | | 19.617 | 0.150 | 19.757 | 0.000 | 1.00000 |
|  | occludin-1 | 26.423 | | 26.519 | | 26.154 | 0.189 | 26.366 | 6.609 | 0.01025 |
| **sample** | **primer** | **CT** | | | | | **SD** | **CT average** | **ΔCT** | **occludin-1/β-actin 2**-**^△^**CT |
| **HK** | β-actin | 22.114 | | 22.096 | | 22.119 | 0.012 | 22.110 | 0.000 | 1.00000 |
|  | occludin-1 | 29.209 | | 28.807 | | 29.055 | 0.203 | 29.023 | 6.914 | 0.00829 |
| **sample** | **primer** | **CT** | | | | | **SD** | **CT average** | **ΔCT** | **occludin-1/β-actin 2**-**^△^**CT |
| **HK** | β-actin | 21.843 | | 21.845 | | 21.953 | 0.063 | 21.880 | 0.000 | 1.00000 |
|  | occludin-1 | 29.701 | | 29.536 | | 29.941 | 0.203 | 29.726 | 7.846 | 0.00435 |
| **sample** | **primer** | **CT** | | | | | **SD** | **CT average** | **ΔCT** | **occludin-1/β-actin 2**-**^△^**CT |
| **RG** | β-actin | 21.012 | | 20.889 | | 21.094 | 0.103 | 20.998 | 0.000 | 1.00000 |
|  | occludin-1 | 24.814 | | 21.940 | | 24.900 | 0.066 | 23.885 | 2.886 | 0.13527 |
| **sample** | **primer** | **CT** | | | | | **SD** | **CT average** | **ΔCT** | **occludin-1/β-actin 2**-**^△^**CT |
| **RG** | β-actin | 22.312 | | 22.129 | | 22.239 | 0.092 | 22.227 | 0.000 | 1.00000 |
|  | occludin-1 | 24.700 | | 24.684 | | 24.579 | 0.066 | 24.655 | 2.428 | 0.18584 |
| **sample** | **primer** | **CT** | | | | | **SD** | **CT average** | **ΔCT** | **occludin-1/β-actin 2**-**^△^**CT |
| **RG** | β-actin | 23.737 | | 24.048 | | 23.938 | 0.157 | 23.907 | 0.000 | 1.00000 |
|  | occludin-1 | 27.970 | | 27.884 | | 28.016 | 0.067 | 27.956 | 4.049 | 0.06041 |
| **sample** | **primer** | **CT** | | | | | **SD** | **CT average** | **ΔCT** | **occludin-1/β-actin 2**-**^△^**CT |
| **RG** | β-actin | 19.765 | | 20.288 | | 19.809 | 0.290 | 19.954 | 0.000 | 1.00000 |
|  | occludin-1 | 24.599 | | 24.961 | | 24.665 | 0.193 | 24.742 | 4.788 | 0.03620 |
| **sample** | **primer** | **CT** | | | | | **SD** | **CT average** | **ΔCT** | **occludin-1/β-actin 2**-**^△^**CT |
| **RG** | β-actin | 22.842 | | 23.335 | | 22.694 | 0.336 | 22.957 | 0.000 | 1.00000 |
|  | occludin-1 | 28.781 | | 27.400 | | 28.642 | 0.761 | 28.274 | 5.317 | 0.02508 |
| **sample** | **primer** | **CT** | | | | | **SD** | **CT average** | **ΔCT** | **occludin-1/β-actin 2**-**^△^**CT |
| **RG** | β-actin | 23.223 | | 23.196 | | 23.311 | 0.060 | 23.243 | 0.000 | 1.00000 |
|  | occludin-1 | 28.612 | | 29.261 | | 29.453 | 0.441 | 29.108 | 5.865 | 0.01715 |
| **sample** | **primer** | **CT** | | | | | **SD** | **CT average** | **ΔCT** | **occludin-1/β-actin 2**-**^△^**CT |
| **CO** | β-actin | 20.652 | | 20.420 | | 20.511 | 0.117 | 20.528 | 0.000 | 1.00000 |
|  | occludin-1 | 24.980 | | 25.374 | | 25.683 | 0.352 | 25.345 | 4.818 | 0.03545 |
| **sample** | **primer** | **CT** | | | | | **SD** | **CT average** | **ΔCT** | **occludin-1/β-actin 2**-**^△^**CT |
| **CO** | β-actin | 22.643 | | 22.613 | | 22.556 | 0.044 | 22.604 | 0.000 | 1.00000 |
|  | occludin-1 | 26.903 | | 26.818 | | 25.989 | 0.505 | 26.570 | 3.966 | 0.06398 |
| **sample** | **primer** | **CT** | | | | | **SD** | **CT average** | **ΔCT** | **occludin-1/β-actin 2**-**^△^**CT |
| **CO** | β-actin | 19.936 | | 19.901 | | 19.858 | 0.039 | 19.898 | 0.000 | 1.00000 |
|  | occludin-1 | 24.530 | | 24.921 | | 24.983 | 0.245 | 24.812 | 4.913 | 0.03319 |
| **sample** | **primer** | **CT** | | | | | **SD** | **CT average** | **ΔCT** | **occludin-1/β-actin 2**-**^△^**CT |
| **CO** | β-actin | 20.935 | | 20.164 | | 20.558 | 0.386 | 20.552 | 0.000 | 1.00000 |
|  | occludin-1 | 27.457 | | 27.670 | | 27.476 | 0.114 | 27.534 | 6.982 | 0.00791 |
| **sample** | **primer** | **CT** | | | | | **SD** | **CT average** | **ΔCT** | **occludin-1/β-actin 2**-**^△^**CT |
| **CO** | β-actin | 18.911 | | 18.795 | | 18.902 | 0.065 | 18.869 | 0.000 | 1.00000 |
|  | occludin-1 | 27.674 | | 27.470 | | 27.404 | 0.141 | 27.516 | 8.647 | 0.00249 |
| **sample** | **primer** | **CT** | | | | | **SD** | **CT average** | **ΔCT** | **occludin-1/β-actin 2**-**^△^**CT |
| **CO** | β-actin | 20.225 | | 20.152 | | 20.565 | 0.221 | 20.314 | 0.000 | 1.00000 |
|  | occludin-1 | 29.935 | | 29.709 | | 29.803 | 0.114 | 29.816 | 9.502 | 0.00138 |
| **sample** | **primer** | **CT** | | | | | **SD** | **CT average** | **ΔCT** | **occludin-1/β-actin 2**-**^△^**CT |
| **RC** | β-actin | 25.320 | | 25.476 | | 25.231 | 0.124 | 25.342 | 0.000 | 1.00000 |
|  | occludin-1 | 30.137 | | 29.429 | | 29.902 | 0.415 | 29.823 | 4.481 | 0.04480 |
| **sample** | **primer** | **CT** | | | | | **SD** | **CT average** | **ΔCT** | **occludin-1/β-actin 2**-**^△^**CT |
| **RC** | β-actin | 23.522 | | 23.566 | | 23.473 | 0.047 | 23.520 | 0.000 | 1.00000 |
|  | occludin-1 | 27.923 | | 28.853 | | 27.181 | 0.838 | 27.986 | 4.465 | 0.04527 |
| **sample** | **primer** | **CT** | | | | | **SD** | **CT average** | **ΔCT** | **occludin-1/β-actin 2**-**^△^**CT |
| **RC** | β-actin | 21.585 | | 21.706 | | 21.652 | 0.061 | 21.648 | 0.000 | 1.00000 |
|  | occludin-1 | 26.803 | | 26.908 | | 26.311 | 0.319 | 26.674 | 5.026 | 0.03069 |
| **sample** | **primer** | **CT** | | | | | **SD** | **CT average** | **ΔCT** | **occludin-1/β-actin 2**-**^△^**CT |
| **RC** | β-actin | 24.496 | | 24.936 | | 24.574 | 0.234 | 24.669 | 0.000 | 1.00000 |
|  | occludin-1 | 29.461 | | 30.276 | | 29.732 | 0.415 | 29.823 | 5.154 | 0.02808 |
| **sample** | **primer** | **CT** | | | | | **SD** | **CT average** | **ΔCT** | **occludin-1/β-actin 2**-**^△^**CT |
| **RC** | β-actin | 21.437 | | 21.466 | | 21.531 | 0.048 | 21.478 | 0.000 | 1.00000 |
|  | occludin-1 | 28.274 | | 27.875 | | 28.110 | 0.201 | 28.086 | 6.608 | 0.01025 |
| **sample** | **primer** | **CT** | | | | | **SD** | **CT average** | **ΔCT** | **occludin-1/β-actin 2**-**^△^**CT |
| **RC** | β-actin | 21.937 | | 21.968 | | 21.971 | 0.019 | 21.959 | 0.000 | 1.00000 |
|  | occludin-1 | 29.384 | | 29.546 | | 29.084 | 0.235 | 29.338 | 7.380 | 0.00600 |
| **sample** | **primer** | **CT** | | | | | **SD** | **CT average** | **ΔCT** | **zo-1/β-actin 2**-**^△^**CT |
| **N** | β-actin | 25.735 | | 25.933 | | 25.836 | 0.099 | 25.835 | 0.000 | 1.00000 |
|  | zo-1 | 26.458 | | 26.625 | | 27.247 | 0.416 | 26.852 | 1.018 | 0.49393 |
| **sample** | **primer** | **CT** | | | | | **SD** | **CT average** | **ΔCT** | **zo-1/β-actin 2**-**^△^**CT |
| **N** | β-actin | 25.711 | | 25.574 | | 25.681 | 0.072 | 25.655 | 0.000 | 1.00000 |
|  | zo-1 | 26.976 | | 25.592 | | 26.876 | 0.772 | 26.926 | 1.271 | 0.41450 |
| **sample** | **primer** | **CT** | | | | | **SD** | **CT average** | **ΔCT** | **zo-1/β-actin 2**-**^△^**CT |
| **N** | β-actin | 22.013 | | 22.110 | | 22.271 | 0.130 | 22.131 | 0.000 | 1.00000 |
|  | zo-1 | 26.610 | | 26.279 | | 26.483 | 0.167 | 26.547 | 4.415 | 0.04686 |
| **sample** | **primer** | **CT** | | | | | **SD** | **CT average** | **ΔCT** | **zo-1/β-actin 2**-**^△^**CT |
| **N** | β-actin | 25.979 | | 25.850 | | 25.897 | 0.065 | 25.909 | 0.000 | 1.00000 |
|  | zo-1 | 27.920 | | 26.401 | | 27.695 | 0.820 | 27.807 | 1.899 | 0.26817 |
| **sample** | **primer** | **CT** | | | | | **SD** | **CT average** | **ΔCT** | **zo-1/β-actin 2**-**^△^**CT |
| **N** | β-actin | 23.796 | | 23.964 | | 23.877 | 0.084 | 23.879 | 0.000 | 1.00000 |
|  | zo-1 | 27.879 | | 27.307 | | 27.968 | 0.359 | 27.923 | 4.044 | 0.06061 |
| **sample** | **primer** | **CT** | | | | | **SD** | **CT average** | **ΔCT** | **zo-1/β-actin 2**-**^△^**CT |
| **N** | β-actin | 18.953 | | 19.003 | | 18.984 | 0.025 | 18.980 | 0.000 | 1.00000 |
|  | zo-1 | 24.042 | | 24.088 | | 24.183 | 0.072 | 24.112 | 5.133 | 0.02851 |
| **sample** | **primer** | **CT** | | | | | **SD** | **CT average** | **ΔCT** | **zo-1/β-actin 2**-**^△^**CT |
| **M** | β-actin | 22.913 | | 22.943 | | 22.941 | 0.017 | 22.932 | 0.000 | 1.00000 |
|  | zo-1 | 26.920 | | 24.695 | | 24.805 | 1.254 | 25.863 | 2.931 | 0.13116 |
| **sample** | **primer** | **CT** | | | | | **SD** | **CT average** | **ΔCT** | **zo-1/β-actin 2**-**^△^**CT |
| **M** | β-actin | 25.048 | | 25.074 | | 24.949 | 0.066 | 25.024 | 0.000 | 1.00000 |
|  | zo-1 | 29.053 | | 28.988 | | 29.339 | 0.187 | 29.196 | 4.173 | 0.05545 |
| **sample** | **primer** | **CT** | | | | | **SD** | **CT average** | **ΔCT** | **zo-1/β-actin 2**-**^△^**CT |
| **M** | β-actin | 20.934 | | 20.762 | | 20.456 | 0.242 | 20.718 | 0.000 | 1.00000 |
|  | zo-1 | 25.917 | | 25.946 | | 25.940 | 0.015 | 25.929 | 5.211 | 0.02700 |
| **sample** | **primer** | **CT** | | | | | **SD** | **CT average** | **ΔCT** | **zo-1/β-actin 2**-**^△^**CT |
| **M** | β-actin | 19.862 | | 19.805 | | 19.908 | 0.051 | 19.858 | 0.000 | 1.00000 |
|  | zo-1 | 24.125 | | 24.292 | | 25.050 | 0.493 | 24.588 | 4.730 | 0.03769 |
| **sample** | **primer** | **CT** | | | | | **SD** | **CT average** | **ΔCT** | **zo-1/β-actin 2**-**^△^**CT |
| **M** | β-actin | 24.050 | | 24.065 | | 24.045 | 0.011 | 24.053 | 0.000 | 1.00000 |
|  | zo-1 | 29.330 | | 29.399 | | 29.366 | 0.035 | 29.348 | 5.295 | 0.02547 |
| **sample** | **primer** | **CT** | | | | | **SD** | **CT average** | **ΔCT** | **zo-1/β-actin 2**-**^△^**CT |
| **M** | β-actin | 17.965 | | 17.950 | | 17.940 | 0.012 | 17.952 | 0.000 | 1.00000 |
|  | zo-1 | 23.836 | | 23.921 | | 23.910 | 0.046 | 23.873 | 5.921 | 0.01650 |
| **sample** | **primer** | **CT** | | | | | **SD** | **CT average** | **ΔCT** | **zo-1/β-actin 2**-**^△^**CT |
| **HK** | β-actin | 25.069 | | 24.991 | | 25.065 | 0.044 | 25.042 | 0.000 | 1.00000 |
|  | zo-1 | 24.840 | | 26.127 | | 24.860 | 0.738 | 24.850 | -0.191 | 1.14194 |
| **sample** | **primer** | **CT** | | | | | **SD** | **CT average** | **ΔCT** | **zo-1/β-actin 2**-**^△^**CT |
| **HK** | β-actin | 25.635 | | 25.569 | | 25.526 | 0.055 | 25.577 | 0.000 | 1.00000 |
|  | zo-1 | 27.744 | | 27.740 | | 27.343 | 0.230 | 27.544 | 1.967 | 0.25574 |
| **sample** | **primer** | **CT** | | | | | **SD** | **CT average** | **ΔCT** | **zo-1/β-actin 2**-**^△^**CT |
| **HK** | β-actin | 19.738 | | 19.916 | | 19.617 | 0.150 | 19.757 | 0.000 | 1.00000 |
|  | zo-1 | 24.926 | | 24.933 | | 24.748 | 0.105 | 24.837 | 5.080 | 0.02956 |
| **sample** | **primer** | **CT** | | | | | **SD** | **CT average** | **ΔCT** | **zo-1/β-actin 2**-**^△^**CT |
| **HK** | β-actin | 22.114 | | 22.096 | | 22.119 | 0.012 | 22.110 | 0.000 | 1.00000 |
|  | zo-1 | 23.976 | | 24.533 | | 24.256 | 0.279 | 24.116 | 2.006 | 0.24895 |
| **sample** | **primer** | **CT** | | | | | **SD** | **CT average** | **ΔCT** | **zo-1/β-actin 2**-**^△^**CT |
| **HK** | β-actin | 21.935 | | 21.455 | | 21.369 | 0.305 | 21.586 | 0.000 | 1.00000 |
|  | zo-1 | 25.778 | | 25.951 | | 26.449 | 0.349 | 26.113 | 4.527 | 0.04336 |
| **sample** | **primer** | **CT** | | | | | **SD** | **CT average** | **ΔCT** | **zo-1/β-actin 2**-**^△^**CT |
| **HK** | β-actin | 21.843 | | 21.845 | | 21.953 | 0.063 | 21.880 | 0.000 | 1.00000 |
|  | zo-1 | 27.375 | | 27.422 | | 27.502 | 0.064 | 27.438 | 5.558 | 0.02122 |
| **sample** | **primer** | **CT** | | | | | **SD** | **CT average** | **ΔCT** | **zo-1/β-actin 2**-**^△^**CT |
| **RG** | β-actin | 23.737 | | 24.048 | | 23.938 | 0.157 | 23.907 | 0.000 | 1.00000 |
|  | zo-1 | 27.462 | | 25.921 | | 27.271 | 0.840 | 27.367 | 3.459 | 0.09091 |
| **sample** | **primer** | **CT** | | | | | **SD** | **CT average** | **ΔCT** | **zo-1/β-actin 2**-**^△^**CT |
| **RG** | β-actin | 23.223 | | 23.196 | | 23.311 | 0.060 | 23.243 | 0.000 | 1.00000 |
|  | zo-1 | 26.882 | | 26.324 | | 26.843 | 0.311 | 26.862 | 3.619 | 0.08138 |
| **sample** | **primer** | **CT** | | | | | **SD** | **CT average** | **ΔCT** | **zo-1/β-actin 2**-**^△^**CT |
| **RG** | β-actin | 19.765 | | 20.288 | | 19.809 | 0.290 | 19.954 | 0.000 | 1.00000 |
|  | zo-1 | 25.156 | | 25.023 | | 25.085 | 0.067 | 25.120 | 5.167 | 0.02784 |
| **sample** | **primer** | **CT** | | | | | **SD** | **CT average** | **ΔCT** | **zo-1/β-actin 2**-**^△^**CT |
| **RG** | β-actin | 22.842 | | 23.335 | | 22.694 | 0.336 | 22.957 | 0.000 | 1.00000 |
|  | zo-1 | 27.092 | | 27.501 | | 26.756 | 0.373 | 26.924 | 3.967 | 0.06395 |
| **sample** | **primer** | **CT** | | | | | **SD** | **CT average** | **ΔCT** | **zo-1/β-actin 2**-**^△^**CT |
| **RG** | β-actin | 22.312 | | 22.129 | | 22.239 | 0.092 | 22.227 | 0.000 | 1.00000 |
|  | zo-1 | 26.524 | | 26.442 | | 26.511 | 0.044 | 26.518 | 4.291 | 0.05109 |
| **sample** | **primer** | **CT** | | | | | **SD** | **CT average** | **ΔCT** | **zo-1/β-actin 2**-**^△^**CT |
| **RG** | β-actin | 21.012 | | 20.889 | | 21.094 | 0.103 | 20.998 | 0.000 | 1.00000 |
|  | zo-1 | 26.675 | | 26.520 | | 26.539 | 0.084 | 26.607 | 5.608 | 0.02050 |
| **sample** | **primer** | **CT** | | | | | **SD** | **CT average** | **ΔCT** | **zo-1/β-actin 2**-**^△^**CT |
| **CO** | β-actin | 22.643 | | 22.613 | | 22.556 | 0.044 | 22.604 | 0.000 | 1.00000 |
|  | zo-1 | 26.307 | | 26.196 | | 26.036 | 0.137 | 26.171 | 3.568 | 0.08434 |
| **sample** | **primer** | **CT** | | | | | **SD** | **CT average** | **ΔCT** | **zo-1/β-actin 2**-**^△^**CT |
| **CO** | β-actin | 20.652 | | 20.420 | | 20.511 | 0.117 | 20.528 | 0.000 | 1.00000 |
|  | zo-1 | 25.700 | | 25.682 | | 25.142 | 0.317 | 25.421 | 4.894 | 0.03364 |
| **sample** | **primer** | **CT** | | | | | **SD** | **CT average** | **ΔCT** | **zo-1/β-actin 2**-**^△^**CT |
| **CO** | β-actin | 20.225 | | 20.152 | | 20.565 | 0.221 | 20.314 | 0.000 | 1.00000 |
|  | zo-1 | 26.696 | | 26.667 | | 26.488 | 0.113 | 26.592 | 6.278 | 0.01289 |
| **sample** | **primer** | **CT** | | | | | **SD** | **CT average** | **ΔCT** | **zo-1/β-actin 2**-**^△^**CT |
| **CO** | β-actin | 19.936 | | 19.901 | | 19.858 | 0.039 | 19.898 | 0.000 | 1.00000 |
|  | zo-1 | 25.480 | | 25.927 | | 25.476 | 0.259 | 25.478 | 5.579 | 0.02091 |
| **sample** | **primer** | **CT** | | | | | **SD** | **CT average** | **ΔCT** | **zo-1/β-actin 2**-**^△^**CT |
| **CO** | β-actin | 20.935 | | 20.164 | | 20.558 | 0.386 | 20.552 | 0.000 | 1.00000 |
|  | zo-1 | 26.527 | | 25.928 | | 25.738 | 0.412 | 26.132 | 5.580 | 0.02090 |
| **sample** | **primer** | **CT** | | | | | **SD** | **CT average** | **ΔCT** | **zo-1/β-actin 2**-**^△^**CT |
| **CO** | β-actin | 18.911 | | 18.795 | | 18.902 | 0.065 | 18.869 | 0.000 | 1.00000 |
|  | zo-1 | 26.874 | | 26.777 | | 26.944 | 0.084 | 26.909 | 8.040 | 0.00380 |
| **sample** | **primer** | **CT** | | | | | **SD** | **CT average** | **ΔCT** | **zo-1/β-actin 2**-**^△^**CT |
| **RC** | β-actin | 25.320 | | 25.476 | | 25.231 | 0.124 | 25.342 | 0.000 | 1.00000 |
|  | zo-1 | 26.919 | | 26.823 | | 26.443 | 0.252 | 26.681 | 1.339 | 0.39542 |
| **sample** | **primer** | **CT** | | | | | **SD** | **CT average** | **ΔCT** | **zo-1/β-actin 2**-**^△^**CT |
| **RC** | β-actin | 24.496 | | 24.936 | | 24.574 | 0.234 | 24.669 | 0.000 | 1.00000 |
|  | zo-1 | 26.250 | | 26.363 | | 26.296 | 0.057 | 26.273 | 1.604 | 0.32891 |
| **sample** | **primer** | **CT** | | | | | **SD** | **CT average** | **ΔCT** | **zo-1/β-actin 2**-**^△^**CT |
| **RC** | β-actin | 21.437 | | 21.466 | | 21.531 | 0.048 | 21.478 | 0.000 | 1.00000 |
|  | zo-1 | 25.757 | | 25.860 | | 25.788 | 0.053 | 25.772 | 4.294 | 0.05097 |
| **sample** | **primer** | **CT** | | | | | **SD** | **CT average** | **ΔCT** | **zo-1/β-actin 2**-**^△^**CT |
| **RC** | β-actin | 23.522 | | 23.566 | | 23.473 | 0.047 | 23.520 | 0.000 | 1.00000 |
|  | zo-1 | 26.023 | | 25.333 | | 26.085 | 0.417 | 26.054 | 2.534 | 0.17271 |
| **sample** | **primer** | **CT** | | | | | **SD** | **CT average** | **ΔCT** | **zo-1/β-actin 2**-**^△^**CT |
| **RC** | β-actin | 21.937 | | 21.968 | | 21.971 | 0.019 | 21.959 | 0.000 | 1.00000 |
|  | zo-1 | 25.167 | | 25.939 | | 26.349 | 0.600 | 25.758 | 3.799 | 0.07182 |
| **sample** | **primer** | **CT** | | | | | **SD** | **CT average** | **ΔCT** | **zo-1/β-actin 2**-**^△^**CT |
| **RC** | β-actin | 21.585 | | 21.706 | | 21.652 | 0.061 | 21.648 | 0.000 | 1.00000 |
|  | zo-1 | 26.538 | | 26.852 | | 26.873 | 0.187 | 26.705 | 5.057 | 0.03003 |

|  | **claudin-1/β-actin 2**-**^△^**CT | **claudin-1/β-actin 2**-**^△△^**CT | **The date of claudin-1/β-actin 2**-**^△△^**CT **is used for statistical chart** |
| --- | --- | --- | --- |
| N | 0.14177 | 1.00000 |  |
|  | 0.09930 | 1.00000 |  |
|  | 0.07365 | 1.00000 |  |
|  | 0.01111 | 1.00000 |  |
|  | 0.01275 | 1.00000 |  |
|  | 0.01031 | 1.00000 |  |
| M | 0.02950 | 0.20804 |  |
|  | 0.01782 | 0.17946 |  |
|  | 0.00683 | 0.09280 |  |
|  | 0.00101 | 0.09075 |  |
|  | 0.00263 | 0.20607 |  |
|  | 0.00076 | 0.07407 |  |
| HK | 0.04758 | 0.33562 |  |
|  | 0.02533 | 0.25511 |  |
|  | 0.01202 | 0.16315 |  |
|  | 0.00283 | 0.25441 |  |
|  | 0.00835 | 0.65481 |  |
|  | 0.00241 | 0.23411 |  |
| RG | 0.01819 | 0.12831 |  |
|  | 0.01463 | 0.14728 |  |
|  | 0.00510 | 0.06927 |  |
|  | 0.00252 | 0.22654 |  |
|  | 0.00392 | 0.30708 |  |
|  | 0.00047 | 0.04546 |  |
| CO | 0.00256 | 0.01804 |  |
|  | 0.00196 | 0.01972 |  |
|  | 0.00149 | 0.02026 |  |
|  | 0.00067 | 0.06061 |  |
|  | 0.00076 | 0.05956 |  |
|  | 0.00062 | 0.06011 |  |
| RC | 0.01607 | 0.11333 |  |
|  | 0.05034 | 0.50689 |  |
|  | 0.02836 | 0.38512 |  |
|  | 0.00583 | 0.52453 |  |
|  | 0.00934 | 0.73252 |  |
|  | 0.00421 | 0.40806 |  |

|  | **occludin-1/β-actin 2^-△^CT** | **occludin-1/β-actin 2^-△△^CT** | **The date of occludin-1/β-actin 2**-**^△△^**CT **is used for statistical chart** |
| --- | --- | --- | --- |
| N | 0.07473 | 1.00000 |  |
|  | 0.10360 | 1.00000 |  |
|  | 0.06239 | 1.00000 |  |
|  | 0.03691 | 1.00000 |  |
|  | 0.01403 | 1.00000 |  |
|  | 0.00870 | 1.00000 |  |
| M | 0.03074 | 0.41130 |  |
|  | 0.03868 | 0.37334 |  |
|  | 0.01385 | 0.22206 |  |
|  | 0.00653 | 0.17698 |  |
|  | 0.00542 | 0.38663 |  |
|  | 0.00154 | 0.17651 |  |
| HK | 0.04094 | 0.54778 |  |
|  | 0.05532 | 0.53398 |  |
|  | 0.02760 | 0.44238 |  |
|  | 0.01025 | 0.27764 |  |
|  | 0.00829 | 0.59124 |  |
|  | 0.00435 | 0.49987 |  |
| RG | 0.13527 | 1.81007 |  |
|  | 0.18584 | 1.79379 |  |
|  | 0.06041 | 0.96826 |  |
|  | 0.03620 | 0.98075 |  |
|  | 0.02508 | 1.78780 |  |
|  | 0.01715 | 1.97233 |  |
| CO | 0.03545 | 0.47442 |  |
|  | 0.06398 | 0.61751 |  |
|  | 0.03319 | 0.53194 |  |
|  | 0.00791 | 0.21434 |  |
|  | 0.00249 | 0.17784 |  |
|  | 0.00138 | 0.15861 |  |
| RC | 0.04480 | 0.59942 |  |
|  | 0.04527 | 0.43693 |  |
|  | 0.03069 | 0.49189 |  |
|  | 0.02808 | 0.76072 |  |
|  | 0.01025 | 0.73064 |  |
|  | 0.00600 | 0.69044 |  |
|  | **zo-1/β-actin 2**-**^△^**CT | **zo-1/β-actin 2**-**^△△^**CT | **The date of zo-1/β-actin 2**-**^△△^**CT **is used for statistical chart** |
| N | 0.49393 | 1.00000 |  |
|  | 0.41450 | 1.00000 |  |
|  | 0.04686 | 1.00000 |  |
|  | 0.26817 | 1.00000 |  |
|  | 0.06061 | 1.00000 |  |
|  | 0.02851 | 1.00000 |  |
| M | 0.13116 | 0.26555 |  |
|  | 0.05545 | 0.13378 |  |
|  | 0.02700 | 0.57610 |  |
|  | 0.03769 | 0.14056 |  |
|  | 0.02547 | 0.42029 |  |
|  | 0.01650 | 0.57881 |  |
| HK | 1.14194 | 2.31193 |  |
|  | 0.25574 | 0.61698 |  |
|  | 0.02956 | 0.63079 |  |
|  | 0.24895 | 0.92835 |  |
|  | 0.04336 | 0.71544 |  |
|  | 0.02122 | 0.74452 |  |
| RG | 0.09091 | 0.18405 |  |
|  | 0.08138 | 0.19633 |  |
|  | 0.02784 | 0.59412 |  |
|  | 0.06395 | 0.23847 |  |
|  | 0.05109 | 0.84289 |  |
|  | 0.02050 | 0.71916 |  |
| CO | 0.08434 | 0.17074 |  |
|  | 0.03364 | 0.08116 |  |
|  | 0.01289 | 0.27502 |  |
|  | 0.02091 | 0.07799 |  |
|  | 0.02090 | 0.34491 |  |
|  | 0.00380 | 0.13330 |  |
| RC | 0.39542 | 0.80056 |  |
|  | 0.32891 | 0.79351 |  |
|  | 0.05097 | 1.08769 |  |
|  | 0.17271 | 0.64404 |  |
|  | 0.07182 | 1.18498 |  |
|  | 0.03003 | 1.05344 |  |
