# Supplementary material for: Preventive effects of the Rehmannia glutinosa Libosch and Cornus officinalis Sieb herb couple on chronic kidney disease rats via modulating the intestinal microbiota and enhancing the intestinal barrier
Source: Front Pharmacol. 2022 Sep 8;13:942032. doi: 10.3389/fphar.2022.942032 (PMC9495080; doi:10.3389/fphar.2022.942032)
Supplement: Supplementary file 2 [file Table2.DOCX]

| Semiquantitative scores assessed by histological analysis of kidney tissue sections from difffferent groups. | | | | |
| --- | --- | --- | --- | --- |
|  |  | kidney | | |
| groups |  | Tubular degeneration and atrophy | Renal tubular crystal | Fibrous tissue hyperplasia |
| **N** | **1** | 0 | 0 | 0 |
|  | **2** | 0 | 0 | 0 |
|  | **3** | 0 | 0 | 0 |
|  | **4** | 0 | 0 | 0 |
|  | **5** | 0 | 0 | 0 |
|  | **6** | 0 | 0 | 0 |
| **M** | **1** | 4 | 3 | 4 |
|  | **2** | 4 | 4 | 4 |
|  | **3** | 4 | 4 | 4 |
|  | **4** | 3 | 4 | 3 |
|  | **5** | 4 | 4 | 4 |
|  | **6** | 4 | 4 | 4 |
| **HK** | **1** | 2 | 2 | 2 |
|  | **2** | 3 | 3 | 3 |
|  | **3** | 2 | 2 | 2 |
|  | **4** | 2 | 2 | 2 |
|  | **5** | 3 | 3 | 2 |
|  | **6** | 2 | 2 | 3 |
| **RG** | **1** | 3 | 2 | 3 |
|  | **2** | 3 | 3 | 3 |
|  | **3** | 3 | 2 | 2 |
|  | **4** | 3 | 2 | 2 |
|  | **5** | 3 | 3 | 3 |
|  | **6** | 2 | 2 | 3 |
| **CO** | **1** | 4 | 4 | 4 |
|  | **2** | 4 | 2 | 4 |
|  | **3** | 4 | 3 | 2 |
|  | **4** | 3 | 2 | 3 |
|  | **5** | 3 | 3 | 2 |
|  | **6** | 2 | 3 | 2 |
| **RC** | **1** | 2 | 2 | 2 |
|  | **2** | 2 | 3 | 2 |
|  | **3** | 3 | 2 | 2 |
|  | **4** | 2 | 2 | 3 |
|  | **5** | 2 | 3 | 2 |
|  | **6** | 3 | 2 | 3 |
|  | According to the severity of the lesions, the semi quantitation was extremely mild "1", mild "2", moderate "3", severe "4", and no lesion was marked as "0" | | | |
