# Supplementary material for: Preventive effects of the Rehmannia glutinosa Libosch and Cornus officinalis Sieb herb couple on chronic kidney disease rats via modulating the intestinal microbiota and enhancing the intestinal barrier
Source: Front Pharmacol. 2022 Sep 8;13:942032. doi: 10.3389/fphar.2022.942032 (PMC9495080; doi:10.3389/fphar.2022.942032)
Supplement: Supplementary file 3 [file DataSheet1.PDF]

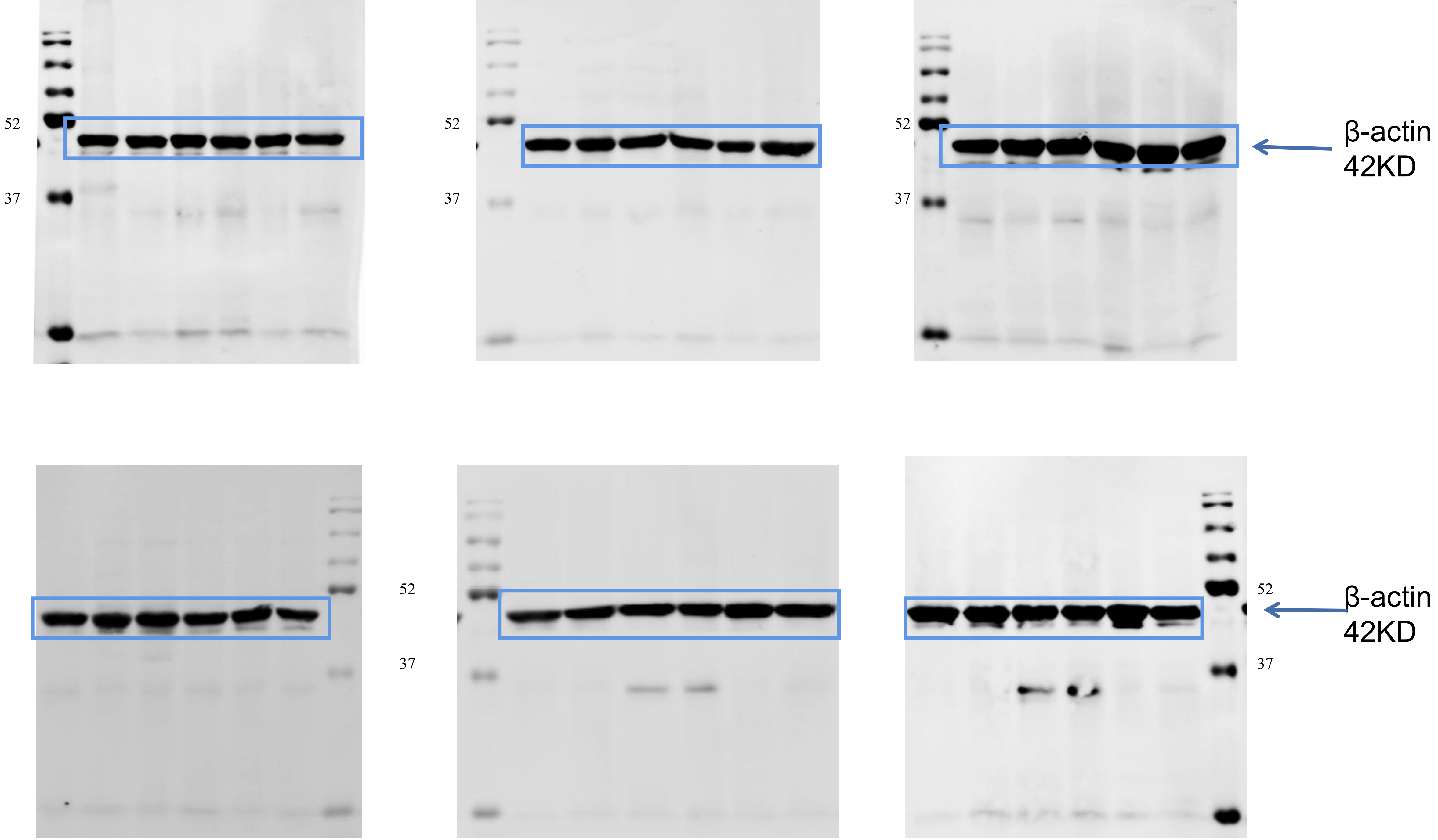
52
37
52
37
52
37
β-actin
42KD
52
37
52
37
β-actin
42KD

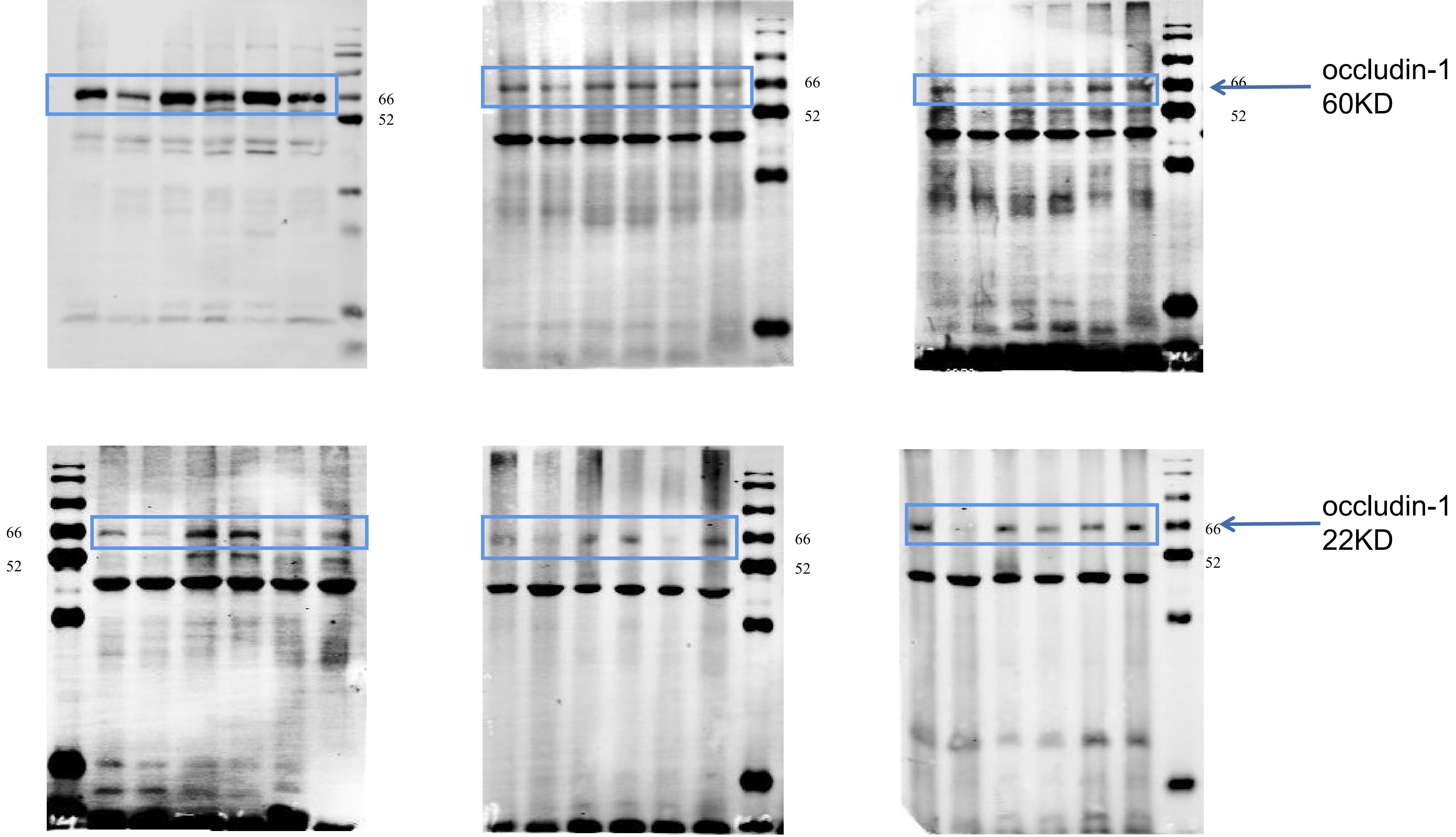
66
52
66
52
66
52
occludin-1
60KD
66
52
66
52
66
52
occludin-1
22KD

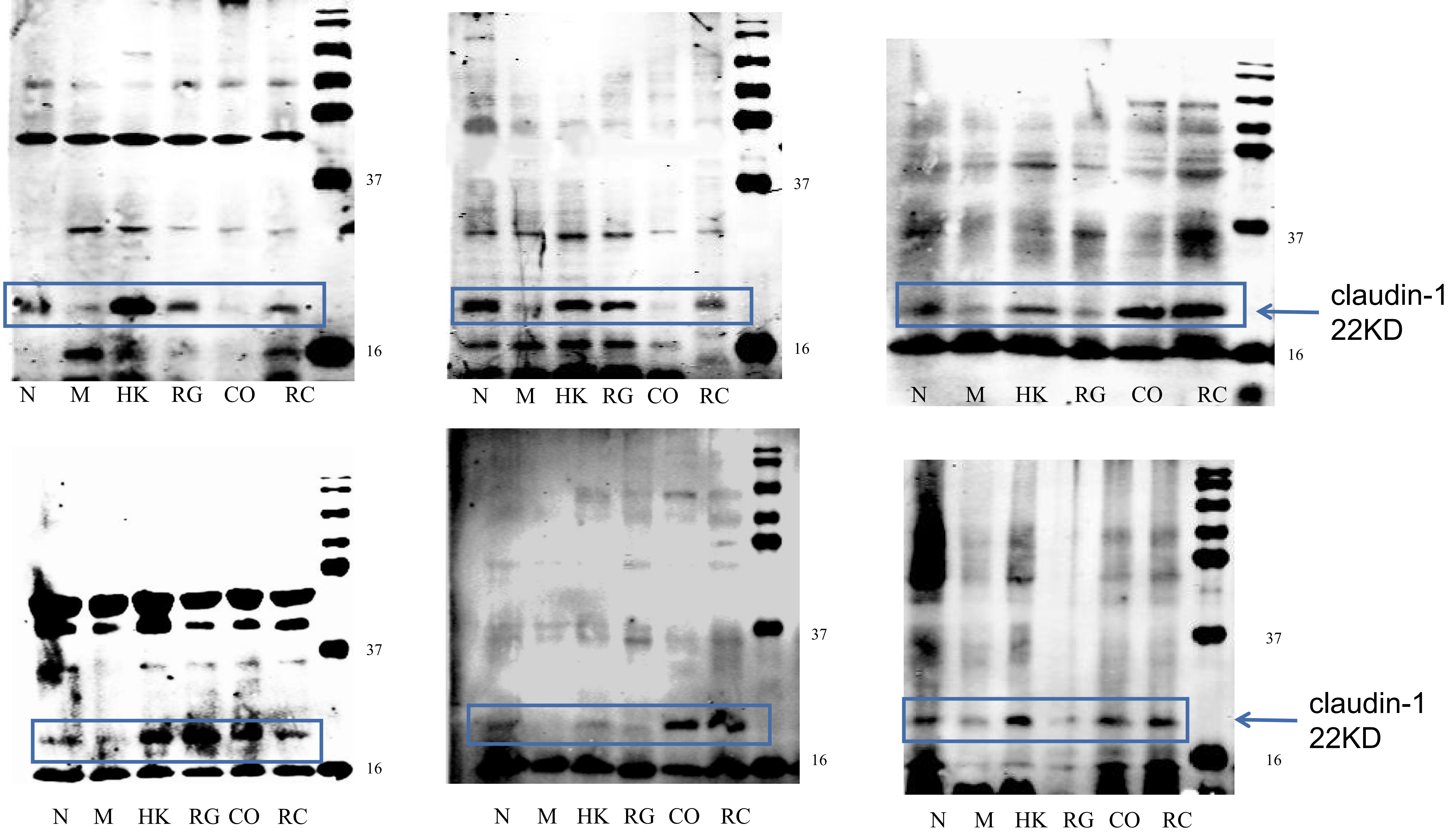
37
16
N M HK RG CO RC
37
16
N M HK RG CO RC
37
16
claudin-1
22KD
N M HK RG CO RC
37
16
N M HK RG CO RC
37
16
N M HK RG CO RC
37
16
claudin-1
22KD
N M HK RG CO RC

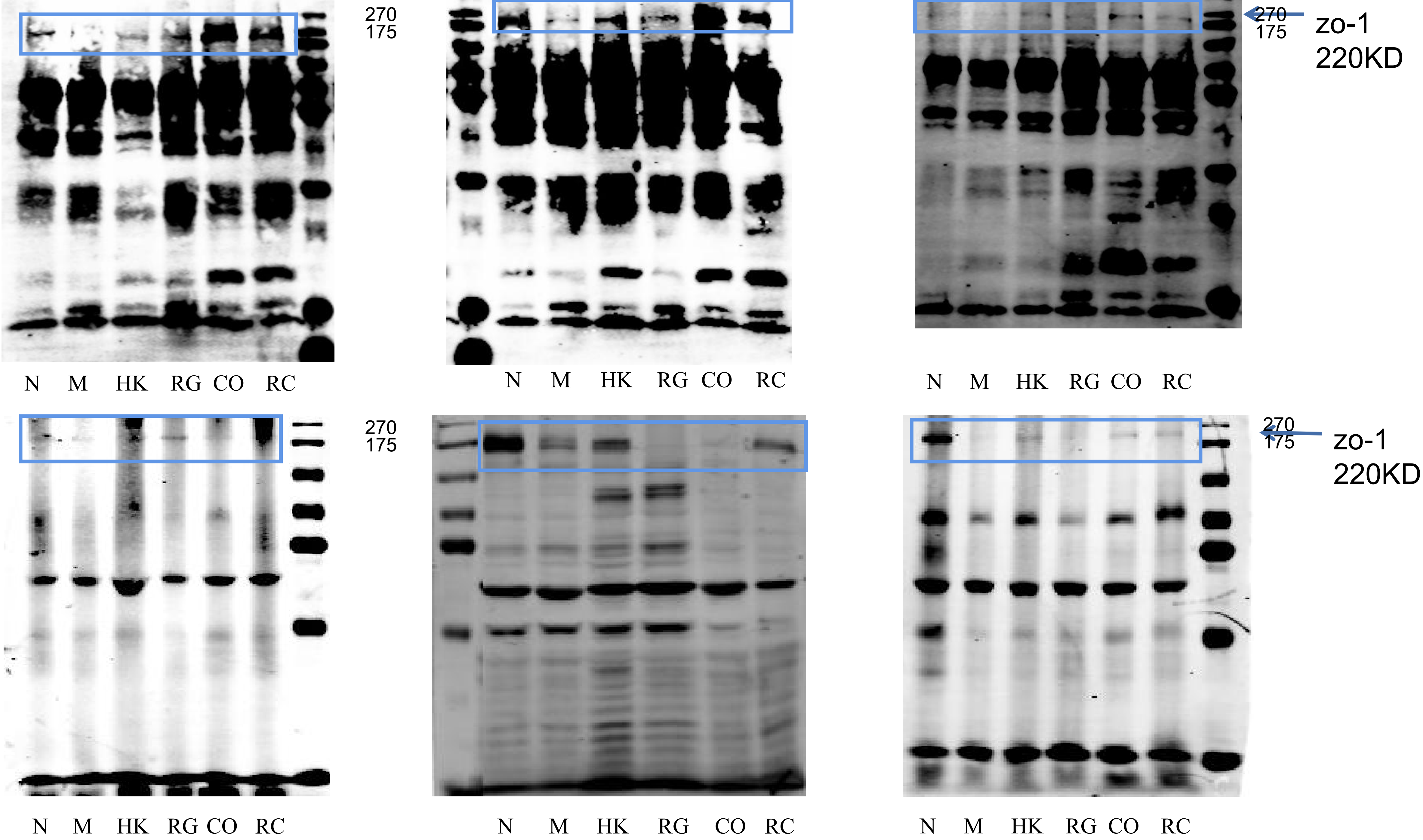
270
175
zo-1
220KD
N M HK RG CO RC
N M HK RG CO RC
N M HK RG CO RC
270
175
zo-1
220KD
N M HK RG CO RC
N M HK RG CO RC
N M HK RG CO RC
