# Supplementary material for: Preventive effects of the Rehmannia glutinosa Libosch and Cornus officinalis Sieb herb couple on chronic kidney disease rats via modulating the intestinal microbiota and enhancing the intestinal barrier
Source: Front Pharmacol. 2022 Sep 8;13:942032. doi: 10.3389/fphar.2022.942032 (PMC9495080; doi:10.3389/fphar.2022.942032)
Supplement: Supplementary file 4 [file Table5.DOCX]

| TER, Ω/cm^2^ | | | | | | |
| --- | --- | --- | --- | --- | --- | --- |
|  | N | M | HK | RG | CO | RC |
| 1 | 58.05 | 46.19 | 50.92 | 48.35 | 49.58 | 53.01 |
| 2 | 57.14 | 46.73 | 52.19 | 47.74 | 48.67 | 52.67 |
| 3 | 58.53 | 44.11 | 50.15 | 48.77 | 49.49 | 53.26 |
| 4 | 56.91 | 45.62 | 51.17 | 47.65 | 49.13 | 53.32 |
| 5 | 57.93 | 44.78 | 52.54 | 48.06 | 48.78 | 52.87 |
| 6 | 57.1 | 45.71 | 51.35 | 48.21 | 48.75 | 52.28 |
|  |  |  |  |  |  |  |
| FD4flux, μg/cm^-2^/h^-1^ | | | | | | |
|  | N | M | HK | RG | CO | RC |
| 1 | 2.703 | 4.358 | 3.752 | 4.042 | 3.528 | 3.508 |
| 2 | 2.687 | 4.216 | 3.761 | 4.136 | 3.464 | 3.485 |
| 3 | 2.694 | 4.498 | 3.723 | 4.079 | 3.507 | 3.517 |
| 4 | 2.651 | 4.298 | 3.745 | 4.115 | 3.475 | 3.499 |
| 5 | 2.728 | 4.387 | 3.736 | 4.098 | 3.514 | 3.532 |
| 6 | 2.675 | 4.237 | 3.691 | 4.024 | 3.492 | 3.477 |
