# Supplementary material for: Preventive effects of the Rehmannia glutinosa Libosch and Cornus officinalis Sieb herb couple on chronic kidney disease rats via modulating the intestinal microbiota and enhancing the intestinal barrier
Source: Front Pharmacol. 2022 Sep 8;13:942032. doi: 10.3389/fphar.2022.942032 (PMC9495080; doi:10.3389/fphar.2022.942032)
Supplement: Supplementary file 5 [file Table3.DOCX]

| ***Butyricimonas*** | | | | | | | |
| --- | --- | --- | --- | --- | --- | --- | --- |
|  |  | **N** | **M** | **HK** | **RG** | **CO** | **RC** |
| **Relative abundance** | **1** | 0.012 | 0.005 | 0.01 | 0.007 | 0.008 | 0.009 |
|  | **2** | 0.014 | 0.007 | 0.011 | 0.009 | 0.009 | 0.012 |
|  | **3** | 0.016 | 0.007 | 0.013 | 0.01 | 0.009 | 0.012 |
|  | **4** | 0.012 | 0.008 | 0.009 | 0.009 | 0.01 | 0.009 |
|  | **5** | 0.014 | 0.005 | 0.011 | 0.012 | 0.011 | 0.013 |
|  | **6** | 0.015 | 0.006 | 0.01 | 0.009 | 0.009 | 0.011 |
|  |  |  |  |  |  |  |  |
| ***Bifidobacterium*** | | | | | | | |
|  |  | **N** | **M** | **HK** | **RG** | **CO** | **RC** |
| **Relative abundance** | **1** | 0.142 | 0.105 | 0.13 | 0.117 | 0.138 | 0.108 |
|  | **2** | 0.144 | 0.087 | 0.111 | 0.139 | 0.109 | 0.152 |
|  | **3** | 0.146 | 0.047 | 0.133 | 0.12 | 0.149 | 0.112 |
|  | **4** | 0.142 | 0.048 | 0.139 | 0.129 | 0.1 | 0.129 |
|  | **5** | 0.144 | 0.025 | 0.101 | 0.152 | 0.141 | 0.133 |
|  | **6** | 0.145 | 0.065 | 0.107 | 0.124 | 0.129 | 0.13 |
|  |  |  |  |  |  |  |  |
| ***Lactobacillus*** | | | | | | | |
|  |  | **N** | **M** | **HK** | **RG** | **CO** | **RC** |
| **Relative abundance** | **1** | 0.212 | 0.105 | 0.187 | 0.167 | 0.138 | 0.208 |
|  | **2** | 0.224 | 0.137 | 0.211 | 0.149 | 0.169 | 0.182 |
|  | **3** | 0.196 | 0.177 | 0.203 | 0.182 | 0.169 | 0.172 |
|  | **4** | 0.232 | 0.138 | 0.193 | 0.179 | 0.201 | 0.209 |
|  | **5** | 0.194 | 0.105 | 0.161 | 0.152 | 0.171 | 0.183 |
|  | **6** | 0.218 | 0.141 | 0.188 | 0.164 | 0.177 | 0.179 |
|  |  |  |  |  |  |  |  |
| ***Lactococcus*** | | | | | | | |
|  |  | **N** | **M** | **HK** | **RG** | **CO** | **RC** |
| **Relative abundance** | **1** | 0.202 | 0.105 | 0.187 | 0.167 | 0.168 | 0.198 |
|  | **2** | 0.214 | 0.141 | 0.21 | 0.169 | 0.158 | 0.202 |
|  | **3** | 0.206 | 0.137 | 0.183 | 0.152 | 0.169 | 0.182 |
|  | **4** | 0.232 | 0.145 | 0.193 | 0.179 | 0.201 | 0.199 |
|  | **5** | 0.199 | 0.105 | 0.161 | 0.152 | 0.171 | 0.187 |
|  | **6** | 0.21 | 0.121 | 0.188 | 0.16 | 0.169 | 0.191 |
|  |  |  |  |  |  |  |  |
| ***Roseburia*** | | | | | | | |
|  |  | **N** | **M** | **HK** | **RG** | **CO** | **RC** |
| **Relative abundance** | **1** | 0.012 | 0.005 | 0.007 | 0.007 | 0.008 | 0.012 |
|  | **2** | 0.009 | 0.001 | 0.007 | 0.009 | 0.008 | 0.01 |
|  | **3** | 0.006 | 0.001 | 0.003 | 0.002 | 0.009 | 0.009 |
|  | **4** | 0.012 | 0.005 | 0.007 | 0.006 | 0.007 | 0.01 |
|  | **5** | 0.009 | 0.003 | 0.011 | 0.012 | 0.01 | 0.007 |
|  | **6** | 0.006 | 0.004 | 0.008 | 0.007 | 0.008 | 0.008 |
|  |  |  |  |  |  |  |  |
| ***Clostridiales*** | | | | | | | |
|  |  | **N** | **M** | **HK** | **RG** | **CO** | **RC** |
| **Relative abundance** | **1** | 0.015 | 0.005 | 0.009 | 0.007 | 0.008 | 0.009 |
|  | **2** | 0.009 | 0.002 | 0.007 | 0.009 | 0.008 | 0.01 |
|  | **3** | 0.01 | 0.001 | 0.006 | 0.005 | 0.009 | 0.008 |
|  | **4** | 0.013 | 0.005 | 0.009 | 0.006 | 0.007 | 0.007 |
|  | **5** | 0.009 | 0.003 | 0.011 | 0.012 | 0.01 | 0.007 |
|  | **6** | 0.009 | 0.004 | 0.008 | 0.008 | 0.007 | 0.007 |
|  |  |  |  |  |  |  |  |
| ***Lachnospiraceae*** | | | | | | | |
|  |  | **N** | **M** | **HK** | **RG** | **CO** | **RC** |
| **Relative abundance** | **1** | 0.315 | 0.185 | 0.229 | 0.207 | 0.208 | 0.209 |
|  | **2** | 0.309 | 0.182 | 0.257 | 0.189 | 0.198 | 0.201 |
|  | **3** | 0.31 | 0.171 | 0.286 | 0.195 | 0.179 | 0.208 |
|  | **4** | 0.313 | 0.185 | 0.199 | 0.206 | 0.201 | 0.207 |
|  | **5** | 0.309 | 0.193 | 0.201 | 0.199 | 0.201 | 0.207 |
|  | **6** | 0.311 | 0.188 | 0.245 | 0.202 | 0.199 | 0.206 |

| ***Phascolarctobacterium*** | | | | | | | |
| --- | --- | --- | --- | --- | --- | --- | --- |
|  |  | **N** | **M** | **HK** | **RG** | **CO** | **RC** |
| **Relative abundance** | **1** | 0.022 | 0.105 | 0.061 | 0.077 | 0.078 | 0.069 |
|  | **2** | 0.024 | 0.187 | 0.058 | 0.079 | 0.079 | 0.062 |
|  | **3** | 0.036 | 0.101 | 0.053 | 0.61 | 0.069 | 0.062 |
|  | **4** | 0.022 | 0.078 | 0.059 | 0.069 | 0.061 | 0.069 |
|  | **5** | 0.019 | 0.085 | 0.061 | 0.05 | 0.051 | 0.063 |
|  | **6** | 0.025 | 0.113 | 0.057 | 0.064 | 0.063 | 0.061 |
|  |  |  |  |  |  |  |  |
| ***Escherichia-Shigella*** | | | | | | | |
|  |  | **N** | **M** | **HK** | **RG** | **CO** | **RC** |
| **Relative abundance** | **1** | 0.002 | 0.009 | 0.006 | 0.007 | 0.008 | 0.005 |
|  | **2** | 0.004 | 0.01 | 0.008 | 0.008 | 0.009 | 0.007 |
|  | **3** | 0.003 | 0.007 | 0.004 | 0.006 | 0.006 | 0.006 |
|  | **4** | 0.002 | 0.008 | 0.007 | 0.006 | 0.001 | 0.006 |
|  | **5** | 0.004 | 0.008 | 0.006 | 0.007 | 0.005 | 0.006 |
|  | **6** | 0.003 | 0.009 | 0.005 | 0.006 | 0.007 | 0.007 |
|  |  |  |  |  |  |  |  |
| ***Bacteroides*** | | | | | | | |
|  |  | **N** | **M** | **HK** | **RG** | **CO** | **RC** |
| **Relative abundance** | **1** | 0.02 | 0.09 | 0.06 | 0.07 | 0.08 | 0.07 |
|  | **2** | 0.04 | 0.1 | 0.06 | 0.08 | 0.08 | 0.07 |
|  | **3** | 0.03 | 0.07 | 0.04 | 0.06 | 0.06 | 0.05 |
|  | **4** | 0.02 | 0.08 | 0.06 | 0.06 | 0.05 | 0.06 |
|  | **5** | 0.04 | 0.09 | 0.06 | 0.07 | 0.05 | 0.06 |
|  | **6** | 0.03 | 0.08 | 0.05 | 0.06 | 0.06 | 0.06 |
|  |  |  |  |  |  |  |  |
| ***Enterococcus*** | | | | | | | |
|  |  | **N** | **M** | **HK** | **RG** | **CO** | **RC** |
| **Relative abundance** | 1 | 0.006 | 0.009 | 0.006 | 0.007 | 0.008 | 0.006 |
|  | 2 | 0.004 | 0.01 | 0.008 | 0.008 | 0.009 | 0.007 |
|  | 3 | 0.005 | 0.011 | 0.007 | 0.006 | 0.006 | 0.007 |
|  | 4 | 0.005 | 0.011 | 0.007 | 0.006 | 0.004 | 0.006 |
|  | 5 | 0.004 | 0.011 | 0.006 | 0.007 | 0.005 | 0.006 |
|  |  | 0.005 | 0.009 | 0.007 | 0.007 | 0.007 | 0.007 |
|  |  |  |  |  |  |  |  |
| ***Faecalibaculum*** | | | | | | | |
|  |  | **N** | **M** | **HK** | **RG** | **CO** | **RC** |
| **Relative abundance** | **1** | 0.012 | 0.029 | 0.02 | 0.023 | 0.024 | 0.022 |
|  | **2** | 0.014 | 0.021 | 0.018 | 0.019 | 0.018 | 0.016 |
|  | **3** | 0.015 | 0.03 | 0.022 | 0.026 | 0.025 | 0.019 |
|  | **4** | 0.009 | 0.021 | 0.017 | 0.018 | 0.015 | 0.013 |
|  | **5** | 0.014 | 0.031 | 0.026 | 0.027 | 0.025 | 0.02 |
|  | **6** | 0.013 | 0.027 | 0.02 | 0.022 | 0.021 | 0.017 |
|  |  |  |  |  |  |  |  |
| ***Romboutsia*** | | | | | | | |
|  |  | **N** | **M** | **HK** | **RG** | **CO** | **RC** |
| **Relative abundance** | **1** | 0.12 | 0.29 | 0.16 | 0.19 | 0.18 | 0.17 |
|  | **2** | 0.14 | 0.29 | 0.16 | 0.18 | 0.18 | 0.12 |
|  | **3** | 0.13 | 0.25 | 0.15 | 0.2 | 0.16 | 0.15 |
|  | **4** | 0.1 | 0.26 | 0.16 | 0.19 | 0.15 | 0.1 |
|  | **5** | 0.14 | 0.23 | 0.16 | 0.17 | 0.15 | 0.16 |
|  | **6** | 0.12 | 0.27 | 0.16 | 0.18 | 0.17 | 0.14 |
|  |  |  |  |  |  |  |  |
| ***Erysipelotrichaceae*** | | | | | | | |
|  |  | **N** | **M** | **HK** | **RG** | **CO** | **RC** |
| **Relative abundance** | **1** | 0.15 | 0.31 | 0.16 | 0.19 | 0.18 | 0.17 |
|  | **2** | 0.18 | 0.29 | 0.23 | 0.2 | 0.18 | 0.15 |
|  | **3** | 0.13 | 0.25 | 0.18 | 0.2 | 0.21 | 0.17 |
|  | **4** | 0.2 | 0.24 | 0.19 | 0.19 | 0.21 | 0.2 |
|  | **5** | 0.17 | 0.28 | 0.2 | 0.21 | 0.24 | 0.2 |
|  | **6** | 0.18 | 0.28 | 0.19 | 0.19 | 0.19 | 0.17 |
