# Supplementary material for: Preventive effects of the Rehmannia glutinosa Libosch and Cornus officinalis Sieb herb couple on chronic kidney disease rats via modulating the intestinal microbiota and enhancing the intestinal barrier
Source: Front Pharmacol. 2022 Sep 8;13:942032. doi: 10.3389/fphar.2022.942032 (PMC9495080; doi:10.3389/fphar.2022.942032)
Supplement: Supplementary file 6 [file Table4.DOCX]

|  | UP |  | Curve formula | A | B | R^2^ |  |  |
| --- | --- | --- | --- | --- | --- | --- | --- | --- |
|  |  |  | Y=A*X+B | 0.013 | -0.089 | 1.000 |  |  |
|  |  |  | N | M | HK | RG | CO | RC |
|  | A | 0.000 | 0.010 | 0.273 | 0.136 | 0.166 | 0.015 | 0.003 |
|  | B | -0.031 | 0.067 | 0.206 | 0.096 | 0.169 | 0.003 | -0.038 |
|  | C | 0.035 | 0.091 | 0.147 | 0.119 | 0.101 | 0.021 | -0.016 |
|  | D | 0.018 | 0.004 | 0.156 | 0.015 | 0.116 | 0.033 | -0.007 |
|  | E | 0.420 | 0.006 | 0.229 | 0.038 | 0.106 | 0.010 | 0.002 |
|  | F | 0.933 | 0.073 | 0.176 | 0.145 | 0.157 | 0.038 | -0.032 |
|  |  |  |  |  |  |  |  |  |
| According to the formula of y=ax+b, substitute the above detection value and calculate the detected concentration value X | |  | N | M | HK | RG | CO | RC |
|  |  | 1 | 7.719 | 28.266 | 17.563 | 19.906 | 8.109 | 7.172 |
|  |  | 2 | 12.172 | 23.031 | 14.438 | 20.141 | 7.172 | 3.969 |
|  |  | 3 | 14.047 | 18.422 | 16.234 | 14.828 | 8.578 | 5.688 |
|  |  | 4 | 7.250 | 19.125 | 8.109 | 16.000 | 9.516 | 6.391 |
|  |  | 5 | 7.406 | 24.828 | 9.906 | 15.219 | 7.728 | 7.094 |
|  |  | 6 | 12.641 | 20.688 | 18.266 | 19.203 | 9.891 | 4.438 |
|  |  |  |  |  |  |  |  |  |
| This data is used for statistical chart | mg/L |  | N | M | HK | RG | CO | RC |
|  | The concentration of up in rats can be obtained from the diluted concentration *5 calculated above | 1 | 38.594 | 141.328 | 87.813 | 99.531 | 40.547 | 35.859 |
|  |  | 2 | 60.859 | 115.156 | 72.188 | 100.703 | 35.859 | 19.844 |
|  |  | 3 | 70.234 | 92.109 | 81.172 | 74.141 | 42.891 | 28.438 |
|  |  | 4 | 36.250 | 95.625 | 40.547 | 80.000 | 47.578 | 31.953 |
|  |  | 5 | 37.031 | 124.141 | 49.531 | 76.094 | 38.641 | 35.469 |
|  |  | 6 | 63.203 | 103.438 | 91.328 | 96.016 | 49.456 | 22.188 |
|  |  |  |  |  |  |  |  |  |
|  |  |  |  |  |  |  |  |  |
|  | IFN |  | Curve formula | A | B | R^2^ |  |  |
|  |  |  | Y=A*X+B | 0.00094 | 0.02930 | 0.995 |  |  |
|  |  |  | N | M | HK | RG | CO | RC |
|  | A | 0.000 | 0.439 | 0.447 | 0.402 | 0.247 | 0.227 | 0.323 |
|  | B | 0.172 | 0.367 | 0.494 | 0.301 | 0.258 | 0.295 | 0.294 |
|  | C | 0.250 | 0.219 | 0.456 | 0.168 | 0.237 | 0.325 | 0.290 |
|  | D | 0.595 | 0.369 | 0.444 | 0.200 | 0.385 | 0.211 | 0.300 |
|  | E | 1.249 | 0.330 | 0.454 | 0.393 | 0.373 | 0.221 | 0.241 |
|  | F | 2.236 | 0.373 | 0.405 | 0.400 | 0.306 | 0.198 | 0.305 |
|  |  |  |  |  |  |  |  |  |
|  |  | pg/mL | N | M | HK | RG | CO | RC |
| According to the formula of y=ax+b, substitute the above detection value and calculate the detected concentration value X | | 1 | 437.25 | 445.78 | 397.76 | 232.34 | 210.99 | 313.45 |
|  |  | 2 | 360.41 | 495.94 | 289.97 | 244.08 | 283.56 | 282.50 |
|  |  | 3 | 202.45 | 455.39 | 148.03 | 221.66 | 315.58 | 278.23 |
|  |  | 4 | 362.54 | 442.58 | 182.18 | 379.62 | 193.92 | 288.90 |
|  |  | 5 | 366.81 | 453.26 | 388.15 | 366.81 | 204.59 | 225.93 |
|  |  | 6 | 320.92 | 400.96 | 395.62 | 295.30 | 180.04 | 294.24 |
|  |  |  |  |  |  |  |  |  |
| This data is used for statistical chart |  | ng/mL | N | M | HK | RG | CO | RC |
|  | Multiply the concentration calculated above by the dilution factor 5 to obtain the concentration of up in rats | 1 | 10.931 | 11.145 | 9.944 | 5.808 | 5.275 | 7.836 |
|  |  | 2 | 9.010 | 12.399 | 7.249 | 6.102 | 7.089 | 7.062 |
|  |  | 3 | 5.061 | 11.385 | 3.701 | 5.542 | 7.890 | 6.956 |
|  |  | 4 | 9.064 | 11.065 | 4.554 | 9.490 | 4.848 | 7.223 |
|  |  | 5 | 9.170 | 11.331 | 9.704 | 9.170 | 5.115 | 5.648 |
|  |  | 6 | 8.023 | 10.024 | 9.891 | 7.383 | 4.501 | 7.356 |
|  |  |  |  |  |  |  |  |  |
|  |  |  |  |  |  |  |  |  |
|  | TNF-α |  | Curve formula | A | B | R^2^ |  |  |
|  |  |  | Y=A*X+B | 0.00200 | -0.02810 | 0.99600 |  |  |
|  |  |  | N | M | HK | RG | CO | RC |
|  | A | 0.000 | 0.073 | 0.129 | 0.092 | 0.063 | 0.074 | 0.323 |
|  | B | 0.028 | 0.088 | 0.135 | 0.076 | 0.100 | 0.092 | 0.294 |
|  | C | 0.047 | 0.075 | 0.142 | 0.100 | 0.075 | 0.038 | 0.290 |
|  | D | 0.139 | 0.052 | 0.108 | 0.101 | 0.092 | 0.062 | 0.300 |
|  | E | 0.360 | 0.099 | 0.123 | 0.067 | 0.037 | 0.111 | 0.241 |
|  | F | 0.684 | 0.104 | 0.133 | 0.089 | 0.086 | 0.127 | 0.305 |
|  |  |  |  |  |  |  |  |  |
|  |  |  | N | M | HK | RG | CO | RC |
| According to the formula of y=ax+b, substitute the above detection value and calculate the detected concentration value X | | 1 | 50.550 | 78.550 | 60.050 | 45.550 | 51.050 | 40.050 |
|  |  | 2 | 58.050 | 81.550 | 52.050 | 64.050 | 60.050 | 52.050 |
|  |  | 3 | 51.550 | 85.050 | 64.050 | 51.550 | 33.050 | 60.050 |
|  |  | 4 | 40.050 | 68.050 | 64.550 | 60.050 | 45.050 | 41.050 |
|  |  | 5 | 63.550 | 75.550 | 47.550 | 32.550 | 69.550 | 48.550 |
|  |  | 6 | 66.050 | 80.550 | 58.550 | 57.050 | 77.550 | 54.550 |
|  |  |  |  |  |  |  |  |  |
| This data is used for statistical chart |  | mg/mL | N | M | HK | RG | CO | RC |
|  | Multiply the concentration calculated above by the dilution factor 25 to obtain the concentration of up in rats | 1 | 1.264 | 1.964 | 1.501 | 1.139 | 1.276 | 1.001 |
|  |  | 2 | 1.451 | 2.039 | 1.301 | 1.601 | 1.501 | 1.301 |
|  |  | 3 | 1.289 | 2.126 | 1.601 | 1.289 | 0.826 | 1.501 |
|  |  | 4 | 1.001 | 1.701 | 1.614 | 1.501 | 1.126 | 1.026 |
|  |  | 5 | 1.589 | 1.889 | 1.189 | 0.814 | 1.739 | 1.214 |
|  |  | 6 | 1.651 | 2.014 | 1.464 | 1.426 | 1.939 | 1.364 |
|  |  |  |  |  |  |  |  |  |
|  |  |  |  |  |  |  |  |  |
|  | TGF-β |  | Curve formula | A | B | R^2^ |  |  |
|  |  |  | Y=A*X+B | 0.00435 | -0.04290 | 0.953 |  |  |
|  |  |  | N | M | HK | RG | CO | RC |
|  | A | 0.000 | 0.218 | 0.063 | 0.234 | 0.151 | 0.110 | 0.162 |
|  | B | 0.169 | 0.228 | 0.069 | 0.226 | 0.220 | 0.151 | 0.193 |
|  | C | 0.166 | 0.238 | 0.055 | 0.182 | 0.163 | 0.161 | 0.186 |
|  | D | 0.203 | 0.267 | 0.097 | 0.203 | 0.141 | 0.156 | 0.126 |
|  | E | 0.508 | 0.233 | 0.025 | 0.244 | 0.208 | 0.192 | 0.275 |
|  | F | 1.433 | 0.246 | 0.039 | 0.204 | 0.212 | 0.188 | 0.247 |
|  |  |  |  |  |  |  |  |  |
|  |  | pg/mL | N | M | HK | RG | CO | RC |
| According to the formula of y=ax+b, substitute the above detection value and calculate the detected concentration value X | | 1 | 59.977 | 24.345 | 63.655 | 44.575 | 35.149 | 47.103 |
|  |  | 2 | 62.276 | 25.724 | 61.816 | 60.437 | 44.575 | 54.230 |
|  |  | 3 | 64.575 | 22.506 | 51.701 | 47.333 | 46.874 | 52.621 |
|  |  | 4 | 71.241 | 32.161 | 56.529 | 42.276 | 45.724 | 38.828 |
|  |  | 5 | 63.425 | 15.609 | 65.954 | 57.678 | 54.000 | 73.080 |
|  |  | 6 | 66.414 | 18.828 | 56.759 | 58.598 | 53.080 | 66.644 |
|  |  |  |  |  |  |  |  |  |
| This data is used for statistical chart |  | ng/mL | N | M | HK | RG | CO | RC |
|  | Multiply the concentration calculated above by the dilution factor 25 to obtain the concentration of up in rats | 1 | 1.499 | 0.609 | 1.591 | 1.114 | 0.879 | 1.178 |
|  |  | 2 | 1.557 | 0.643 | 1.545 | 1.511 | 1.114 | 1.356 |
|  |  | 3 | 1.614 | 0.563 | 1.293 | 1.183 | 1.172 | 1.316 |
|  |  | 4 | 1.781 | 0.804 | 1.413 | 1.057 | 1.143 | 0.971 |
|  |  | 5 | 1.586 | 0.390 | 1.649 | 1.442 | 1.350 | 1.827 |
|  |  | 6 | 1.660 | 0.471 | 1.419 | 1.465 | 1.327 | 1.666 |
|  |  |  |  |  |  |  |  |  |
|  |  |  |  |  |  |  |  |  |
|  | IL-4 |  | Curve formula | A | B | R^2^ |  |  |
|  |  |  | Y=A*X+B | 0.00544 | -0.05780 | 0.997 |  |  |
|  |  |  | N | M | HK | RG | CO | RC |
|  | A | 0.000 | 0.123 | 0.112 | 0.194 | 0.131 | 0.152 | 0.133 |
|  | B | -0.007 | 0.137 | 0.111 | 0.147 | 0.127 | 0.139 | 0.137 |
|  | C | 0.029 | 0.144 | 0.122 | 0.154 | 0.124 | 0.122 | 0.139 |
|  | D | 0.102 | 0.155 | 0.121 | 0.207 | 0.125 | 0.098 | 0.136 |
|  | E | 0.247 | 0.142 | 0.115 | 0.139 | 0.123 | 0.125 | 0.124 |
|  | F | 0.606 | 0.151 | 0.111 | 0.199 | 0.129 | 0.163 | 0.129 |
|  |  |  |  |  |  |  |  |  |
|  |  | pg/mL | N | M | HK | RG | CO | RC |
| According to the formula of y=ax+b, substitute the above detection value and calculate the detected concentration value X | | 1 | 33.235 | 31.213 | 46.287 | 34.706 | 38.566 | 35.074 |
|  |  | 2 | 35.809 | 31.029 | 37.647 | 33.971 | 36.176 | 35.809 |
|  |  | 3 | 37.096 | 33.051 | 38.934 | 33.419 | 33.051 | 36.176 |
|  |  | 4 | 39.118 | 32.868 | 48.676 | 33.603 | 28.640 | 35.625 |
|  |  | 5 | 36.728 | 31.765 | 36.176 | 33.235 | 33.603 | 33.419 |
|  |  | 6 | 38.382 | 31.029 | 47.206 | 34.338 | 40.588 | 34.338 |
|  |  |  |  |  |  |  |  |  |
| This data is used for statistical chart |  | pg/mL | N | M | HK | RG | CO | RC |
|  | Multiply the concentration calculated above by the dilution factor 25 to obtain the concentration of up in rats | 1 | 830.88 | 780.33 | 1157.17 | 867.65 | 964.15 | 876.84 |
|  |  | 2 | 895.22 | 775.74 | 941.18 | 849.26 | 904.41 | 895.22 |
|  |  | 3 | 927.39 | 826.29 | 973.35 | 835.48 | 826.29 | 904.41 |
|  |  | 4 | 977.94 | 821.69 | 1216.91 | 840.07 | 715.99 | 890.63 |
|  |  | 5 | 918.20 | 794.12 | 904.41 | 830.88 | 840.07 | 835.48 |
|  |  | 6 | 959.56 | 775.74 | 1180.15 | 858.46 | 1014.71 | 858.46 |
|  |  |  |  |  |  |  |  |  |
|  |  |  |  |  |  |  |  |  |
|  | IL-6 |  | Curve formula | A | B | R^2^ |  |  |
|  |  |  | Y=A*X+B | 0.00358 | 0.06970 | 0.987 |  |  |
|  |  |  | N | M | HK | RG | CO | RC |
|  | A | 0.000 | 0.125 | 0.274 | 0.150 | 0.109 | 0.137 | 0.134 |
|  | B | 0.068 | 0.162 | 0.223 | 0.156 | 0.161 | 0.137 | 0.190 |
|  | C | 0.141 | 0.127 | 0.232 | 0.158 | 0.140 | 0.161 | 0.128 |
|  | D | 0.192 | 0.113 | 0.205 | 0.098 | 0.147 | 0.128 | 0.077 |
|  | E | 0.380 | 0.093 | 0.189 | 0.171 | 0.105 | 0.141 | 0.118 |
|  | F | 0.495 | 0.141 | 0.201 | 0.134 | 0.141 | 0.147 | 0.122 |
|  |  |  |  |  |  |  |  |  |
|  |  | pg/mL | N | M | HK | RG | CO | RC |
| According to the formula of y=ax+b, substitute the above detection value and calculate the detected concentration value X | | 1 | 15.447 | 57.067 | 22.430 | 10.978 | 18.799 | 17.961 |
|  |  | 2 | 25.782 | 42.821 | 24.106 | 25.503 | 18.799 | 33.603 |
|  |  | 3 | 16.006 | 45.335 | 24.665 | 19.637 | 25.503 | 16.285 |
|  |  | 4 | 12.095 | 37.793 | 7.905 | 21.592 | 16.285 | 2.039 |
|  |  | 5 | 6.508 | 33.324 | 28.296 | 9.860 | 19.916 | 13.492 |
|  |  | 6 | 19.916 | 36.676 | 17.961 | 19.916 | 21.592 | 14.609 |
|  |  |  |  |  |  |  |  |  |
| This data is used for statistical chart |  | pg/mL | N | M | HK | RG | CO | RC |
|  | Multiply the concentration calculated above by the dilution factor 25 to obtain the concentration of up in rats | 1 | 386.17 | 1426.68 | 560.75 | 274.44 | 469.97 | 449.02 |
|  |  | 2 | 644.55 | 1070.53 | 602.65 | 637.57 | 469.97 | 840.08 |
|  |  | 3 | 400.14 | 1133.38 | 616.62 | 490.92 | 637.57 | 407.12 |
|  |  | 4 | 302.37 | 944.83 | 197.63 | 539.80 | 407.12 | 50.98 |
|  |  | 5 | 162.71 | 833.10 | 707.40 | 246.51 | 497.91 | 337.29 |
|  |  | 6 | 497.91 | 916.90 | 449.02 | 497.91 | 539.80 | 365.22 |
